# Distinct genetic variation and heterogeneity of the Iranian population

**DOI:** 10.1371/journal.pgen.1008385

**Published:** 2019-09-24

**Authors:** Zohreh Mehrjoo, Zohreh Fattahi, Maryam Beheshtian, Marzieh Mohseni, Hossein Poustchi, Fariba Ardalani, Khadijeh Jalalvand, Sanaz Arzhangi, Zahra Mohammadi, Shahrouz Khoshbakht, Farid Najafi, Pooneh Nikuei, Mohammad Haddadi, Elham Zohrehvand, Morteza Oladnabi, Akbar Mohammadzadeh, Mandana Hadi Jafari, Tara Akhtarkhavari, Ehsan Shamsi Gooshki, Aliakbar Haghdoost, Reza Najafipour, Lisa-Marie Niestroj, Barbara Helwing, Yasmina Gossmann, Mohammad Reza Toliat, Reza Malekzadeh, Peter Nürnberg, Kimia Kahrizi, Hossein Najmabadi, Michael Nothnagel

**Affiliations:** 1 Genetics Research Center, University of Social Welfare and Rehabilitation Sciences, Tehran, Iran; 2 Digestive Disease Research Centre, Digestive Disease Research Institute, Shariati Hospital, Tehran University of Medical Sciences, Tehran, Iran; 3 Research Center for Environmental Determinants of Health, Kermanshah University of Medical Sciences, Kermanshah, Iran; 4 Molecular Medicine Research Center, Hormozgan Health Institute, Hormozgan University of Medical Sciences, Bandar Abbas, Iran; 5 Department of Biology, University of Zabol, Zabol, Iran; 6 Congenital Malformations Research Center, Golestan University of Medical Sciences, Gorgan, Iran; 7 Department of Medical Genetics, Faculty of Advanced Medical Technologies, School of Advanced Technologies in Medicine, Golestan University of Medical Sciences, Gorgan, Iran; 8 Medical Ethics and History of Medicine Research Center, Tehran University of Medical Sciences, Tehran, Iran; 9 Department of Medical Ethics, Faculty of Medicine, Tehran University of Medical Sciences, Tehran, Iran; 10 Modeling in Health Research Center, Institute for Futures Studies in Health, Kerman University of Medical Sciences, Kerman, Iran; 11 Regional Knowledge Hub, and WHO Collaborating Centre for HIV Surveillance, Institute for Futures Studies in Health, Kerman University of Medical Sciences, Kerman, Iran; 12 Cellular and Molecular Research Centre, Genetic Department, Qazvin University of Medical Sciences, Qazvin, Iran; 13 Cologne Center for Genomics, University of Cologne, Cologne, Germany; 14 Department of Archaeology, The University of Sydney, Australia; 15 Noncommunicable Disease Research Center, Shiraz University of Medical Science, Shiraz, Iran; 16 University Hospital Cologne, Cologne, Germany; University of Pennsylvania, UNITED STATES

## Abstract

Iran, despite its size, geographic location and past cultural influence, has largely been a blind spot for human population genetic studies. With only sparse genetic information on the Iranian population available, we pursued its genome-wide and geographic characterization based on 1021 samples from eleven ethnic groups. We show that Iranians, while close to neighboring populations, present distinct genetic variation consistent with long-standing genetic continuity, harbor high heterogeneity and different levels of consanguinity, fall apart into a cluster of similar groups and several admixed ones and have experienced numerous language adoption events in the past. Our findings render Iran an important source for human genetic variation in Western and Central Asia, will guide adequate study sampling and assist the interpretation of putative disease-implicated genetic variation. Given Iran’s internal genetic heterogeneity, future studies will have to consider ethnic affiliations and possible admixture.

## Author summary

Based on genome-wide genotype data on over 1000 samples from eleven ethnic groups present in Iran and by comparison to reference data sets of both extant populations and ancient DNA samples, we show that the Iranian population comprises distinct genetic variation with respect to populations in close geographic proximity, a cluster of genetically largely overlapping ethnic groups as well as a number of strongly admixed groups. These observations, also corroborated by *f3* migration statistics and other approaches, indicate genetic continuity of and limited influx into the cluster groups over several millennia, despite Iran’s geographic position at a crossroads in West Asia. They also suggest, correspondingly, several instances of language adoption instead of demic replacement in the past. Future human genetic studies, both with a focus on population and medical genetics, will have to consider differences in heterogeneity, consanguinity and degree of admixture between the ethnic groups for an adequate design and interpretation.

## Introduction

The highlands of Iran have been at the crossroads of human migrations [1–6] since the dispersal of modern humans out of Africa due to their geostrategic position. While exercising a strong cultural influence on neighboring regions, Iran has also repeatedly received migratory influx in the past millennia. Among others, this includes the successive southward migration of groups of Indo-European (IE) language speakers (e.g. Scythians, Medes and Persians) [7], the Arab arrival in the 7^th^ century CE and the later influx of Turkic-speaking people from Central Asia. As a result of migrations, internal splits, admixture and other movements, today’s Iranian population comprises numerous ethnic, religious and linguistic groups (S1 Appendix, S1 Fig), prominently including Persians (65% in 2008 [8]), Iranian Azeris (16%), Iranian Kurds (7%), Iranian Lurs (6%), Iranian Arabs (2%), Iranian Baluchis (2%), Iranian Turkmen (1%), Qashqai and other Turkish-language tribal groups (1%) as well as Armenians, Assyrians, Georgians, Jews, Zoroastrians (all <1%) and others, although definitions [9] and reported proportions vary between sources (e.g. [10–12]). Speakers of an Iranian, i.e. Indo-European, language or language dialect (e.g. Persian, Kurdish, Luri, Baluchi) by far outnumber speakers of either a Turkic or Semitic language.

With Iran being located within a belt of countries where consanguineous marriages are widely practiced, Iranian samples have featured prominently in disease-related studies, facilitating the identification of genes involved in rare autosomal recessive diseases by linkage analysis and autozygosity mapping and contributing to a deeper etiological understanding also of complex disorders [13, 14]. Examples demonstrating the value of these populations for human genetic research are ample (e.g. [15–21] for Iran alone), likely moving from the study of few families to population-based studies in the future [22–25]. Still, consanguinity levels are not homogenous across the Iranian population. Early studies, based not on actual genetic data but on familial relation assessment, found these levels to vary between geographic regions and between ethnic groups [26, 27]. A recent study, also based on familial relation assessment, refined these results and reported differences in consanguinity by province, area of residence, birth and marriage cohort as well as with educational level [28]. Patterns of runs of homozygosity (ROHs) or haplotype sharing by descent (HBD) can be indicative of autozygosity, but vary between populations and across genomic locations [29–34], as do the frequency of consanguinity and the moderately correlated degree of genomic inbreeding [35]. Furthermore, autozygosity mapping is predominantly able to detect comparatively recent, local founder mutations [13]. Moreover, carrier frequencies of disease-predisposing variants have been reported to strongly differ between geographic regions in Iran, e.g. for mutations in the *GJB2* gene [36] and for β-thalassemia [37], with different ethnic affiliations being the likely cause and possibly helping to determine the pathogenicity of those variants [38]. Finally, studies on copy-number variation (CNV) in the Iranian population (e.g. [39]) were so far disease-specific but not with respect to the general, healthy population.

Perhaps somewhat surprisingly, Central Asia and parts of Western Asia have largely been a blind spot for non-medical genetic studies in the past decades. Until recently, dedicated genetic projects of extant human populations with a global or continental focus (e.g. [33, 40–51] only sporadically included samples, if any, from Iran and did not comprehensively cover the area. Of note, studies that did include Iranian samples frequently treated them as coming from or being representative of a single homogeneous population.

Studies on sporadic ancient DNA (aDNA) samples from the Early Neolithic up to the Chalcolithic in Iran showed the existence of highly genetically differentiated populations that were not ancestral to Europeans but, in the case of specimen from the Zagros Mountains, exhibited some affinity to Zoroastrians [1, 2, 6, 52]. An early study on ABO blood groups found extreme differences between some of 21 considered ethnic groups in Iran [53], whereas another study, published a year later and additionally based on serum proteins and cell enzymes, presented evidence for population substructure between the six included groups (Iranian Turks, Kurds, Lurs, Zabolis, Baluchis and Zoroastrians) with an average F_ST_ value of 0.02, based on blood groups, serum proteins and cell enzymes, and some degree of inbreeding [54]. More regionally focused studies on Iran, based on uniparental markers such as Y-chromosomal haplogroups and short tandem repeat (STR) marker haplotypes as well as mitochondrial (mtDNA) haplogroups, confirmed high degrees of genetic diversity in the Iranian population [3–5, 55–61]. These studies reported the respective variation to be predominantly of Western Eurasian origin, with only limited contributions from eastern Eurasia, South Asia and Africa most pronounced in the southern Iranian provinces. These studies also reported ancient and recent gene flow between Iran and the Arabian Peninsula, a surprisingly close relationship between Persians and Iranian Turkic-speaking Qashqai and generally high levels of variation comparable to those in the South Caucasus, Anatolia and Europe. These observations all support the notion of Iran forming a crossroads of human migrations. Notably, a study on Armenians, located to the North of Iran, also suggested multiple admixture events and a general role as bridge between different geographic regions [49].

Using genome- or exome-wide genotype data, a number of studies have analyzed samples of populations that can be considered proxies for ethnic groups in Iran from surrounding countries. In a study of 156 individuals, the population of Qatar was reported to comprise three distinct groups, with one (“Q2”) showing strong affinity to Persians and patterns of admixture [3, 62, 63]. A study on 22 Kuwaitis with Persian ancestry found comparatively high levels of genetic diversity for a non-African population, explicable by past admixture events [34]. A study of 43 individuals belonging to the Parsis, a Zoroastrian religious community in India and Pakistan, demonstrated a closer genetic affinity to today’s Iranian and Caucasus populations than to South Asian populations, but, quite remarkably, an even stronger similarity to Neolithic aDNA samples from Iran compared to modern Iranians, consistent both with the historic record of a southward migration induced by the 7^th^ century’s Arab entry to Iran and more recent admixture events with the modern Iranian population [64]. Findings of increased homogeneity and the dating of past admixture events in further samples of Iranian and Indian Zoroastrians [65] complemented these results. Analysis of 24 individuals from the Indo-European speaking Kalash, a population isolate at the Hindu Kush, Afghanistan, indicated a genetically drifted ancient northern Eurasian population that split during the very early Neolithic and subsequently migrated southwards [66]. Finally, a recent study restricted to exome data merged 87 Iranian with 136 Pakistani samples and demonstrated a somewhat extreme or isolated position when compared to other populations from the Maghreb and from the Arabian Peninsula through Turkey [33]. Still, none of these studies has directly and comparatively studied ethnic groups in Iran.

Correlation between genetic and linguistic proximity of populations has frequently been assumed to be the rule, while language adoption is usually considered as an exception to the rule of co-evolution (e.g. [67–69]), although such claims have repeatedly been disputed (e.g. [70]). Evidence for such correlation is ample in Europe, including autosomal and mitochondrial data [71–77], Y-chromosomal data [77–80] and even, with respect to the spread of Indo-European languages into Europe, ancient DNA data [81]. In-depth studies on other parts of the world found some correlation of language dispersal with Y-chromosomal lineages [82–87], although not in all parts [88]. Furthermore, some instances of male-mediated gene flow over major linguistic barriers have been inferred as well [89, 90]. An early study already observed close genetic relationship between Semitic-speaking and Indo-European-speaking groups in Iran [58]. Studies on neighboring Armenia found evidence for a language replacement [91] event, possibly facilitated by the mixing of multiple source populations during the Bronze Age [49]. However, the relationship between genetic and linguistic proximity has been rarely investigated for Iran and neighboring countries.

While Iran appears to be destined to make further important contributions to human genetic research, an adequate design and interpretation of future medical and population genetic studies is mandatory to arrive at interpretable findings. Here, we comprehensively analyzed the genome-wide diversity of eleven ethnic groups in Iran, their relation to each other as well as with global and local reference populations. Furthermore, we investigated, stratified by ethnicity, levels of consanguinity, the distribution of homozygous and copy-number regions and CNVs as well as the extent of population stratification within Iran and the possible effects in association studies if not accounted for properly and the relationship between spoken language family and genetic proximity.

## Results

We compiled a genome-wide data set comprising 1021 unrelated individuals from 11 major Iranian ethnic groups living in Iran (Table 1). For comparison with extant populations, this Iranian data set was merged with either samples from the 1000 Genomes (“1000G”) Project [41–43] (*global data set*) or with those from three recent studies with a more regionalized focus [2, 6, 44] (*local data set*), being further grouped by geographic region (S1 Table) or language family (S2 Table). We also compiled 798 human ancient DNA (aDNA) samples from 21 different publications and one pre-print [2, 6, 81, 92–110] (S3 and S4 Tables) for spatial-temporal analysis.

**Table 1:** Samples included in this study.

Ethnic group	Language subfamily (top-level family)	Samples before QC	Samples after QC (female/male)
Iranian Arabs	Arabic (Afro-Asiatic)	100*	96 (42/54)
Iranian Azeris	Azeric (Turkic)	100*	99 (44/55)
Iranian Baluchis	Iranian (Indo-European)	100*	92 (37/55)
Iranian Gilaks	Iranian (Indo-European)	77	75 (29/46)
Iranian Kurds	Iranian (Indo-European)	100*	97 (46/51)
Iranian Lurs	Iranian (Indo-European)	100*	98 (58/40)
Iranian Mazanderanis (Tabari)	Iranian (Indo-European)	92	87 (38/49)
Iranian Persians	Iranian (Indo-European)	100*	95 (51/44)
Iranian Persian Gulf (PG) Islanders	Iranian (Indo-European)	100*	91 (43/48)
Iranian Sistanis	Iranian (Indo-European)	100	94 (49/45)
Iranian Turkmen	Turkmen (Turkic)	100*	97 (47/50)
Total		1069	1021 (484/537)

Given are ethnic affiliation, spoken language families (according to Glottolog 3.2; http://glottolog.org/) and the number of samples per ethnic group before and after quality control (QC).

*: Samples were part of the Iranome project [124].

### Distinct genetic diversity and substantial heterogeneity

**Fig 1:**
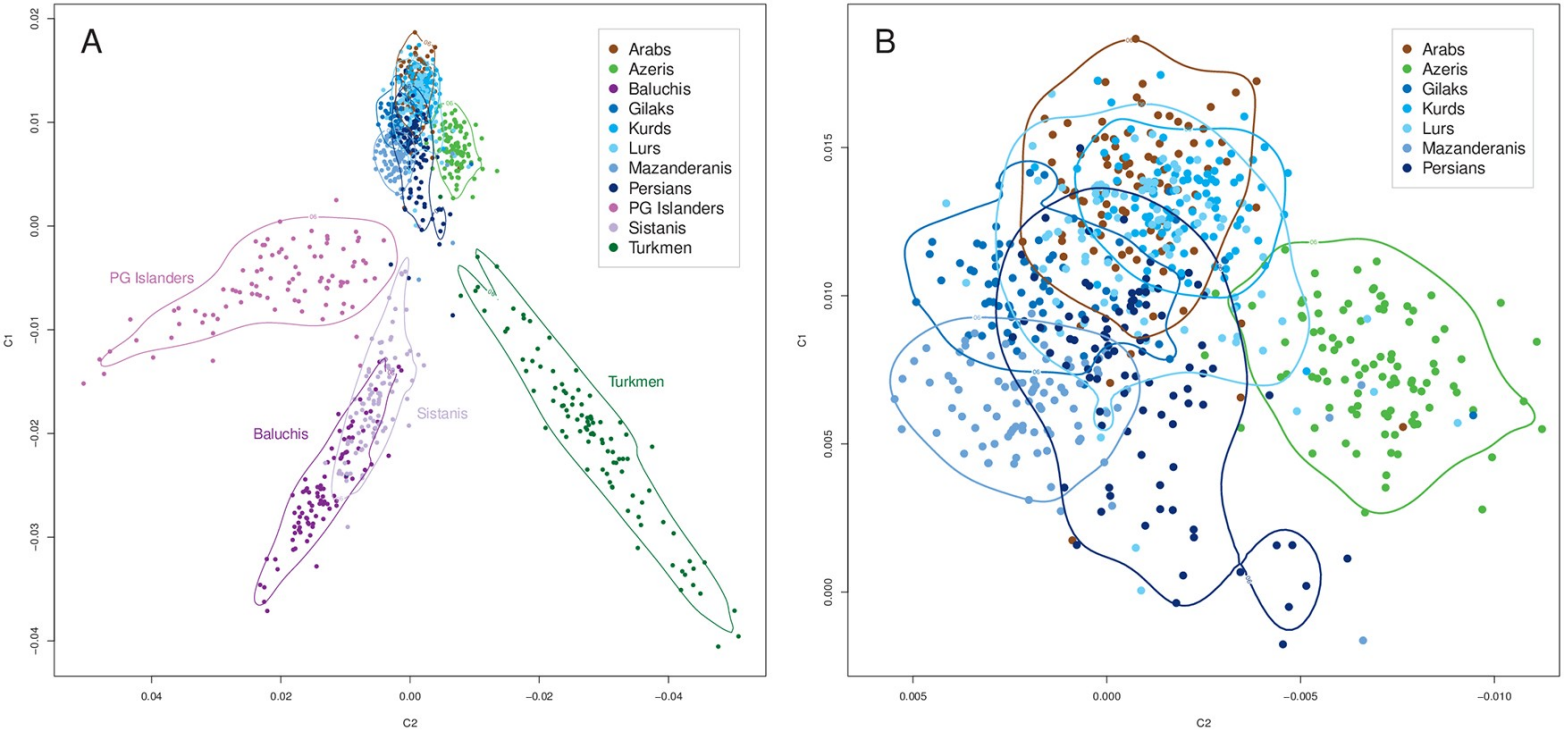
Internal Iranian population structure. Relative sample locations with respect to the first two MDS components. **(A)** Relative sample locations of the Iranian ethnic groups from this study, including 90% density limits; **(B)** zoomed view into the subset of the seven groups belonging to the Central Iranian Cluster (CIC).

**Fig 2:**
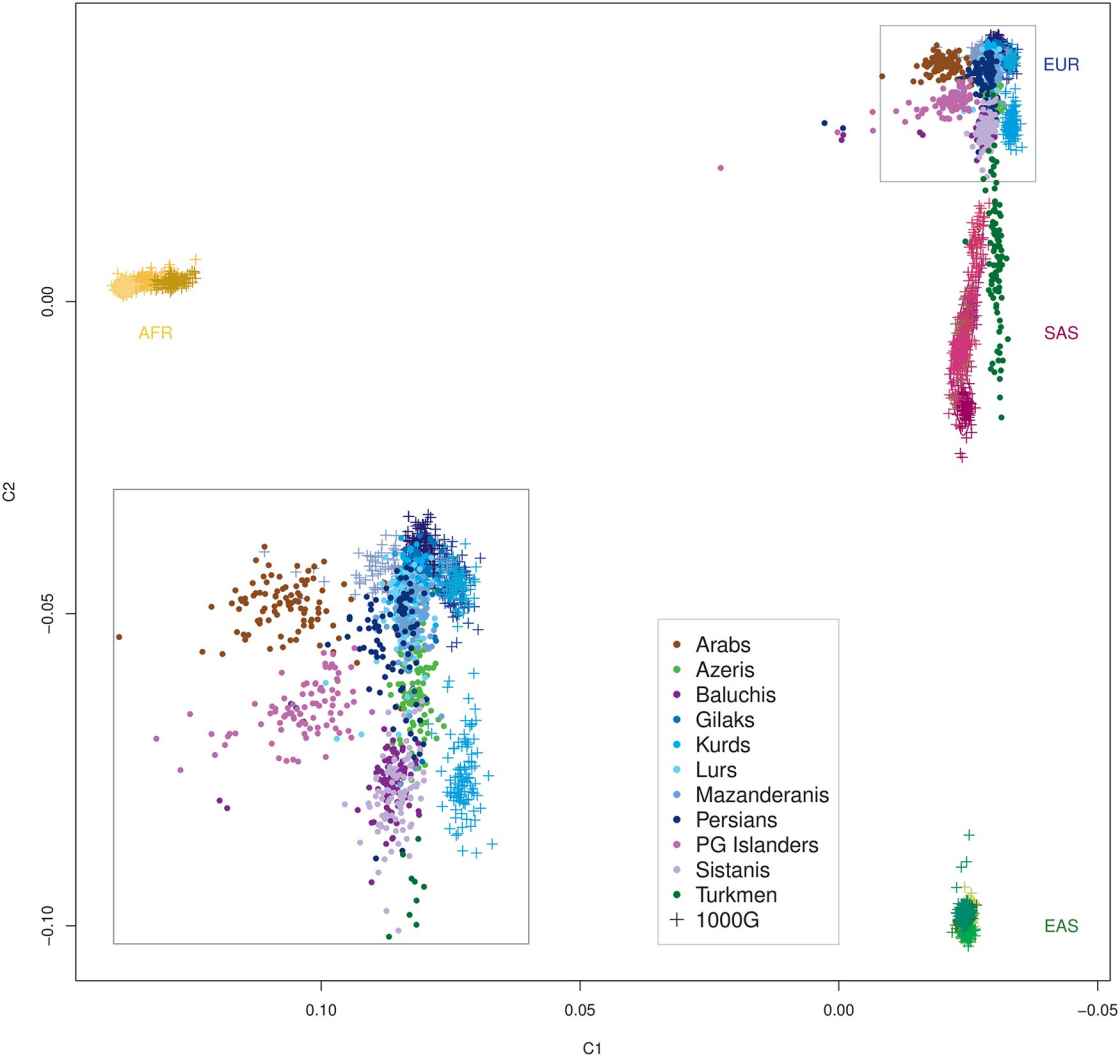
Iranian ethnic groups in a global context. Relative sample locations with respect to the first two MDS components. Iranian ethnic groups in a global context (subset of “Old World” populations from the global 1000G data set); *inlet* zoomed view of the CIC and adjacent European populations.

**Fig 3:**
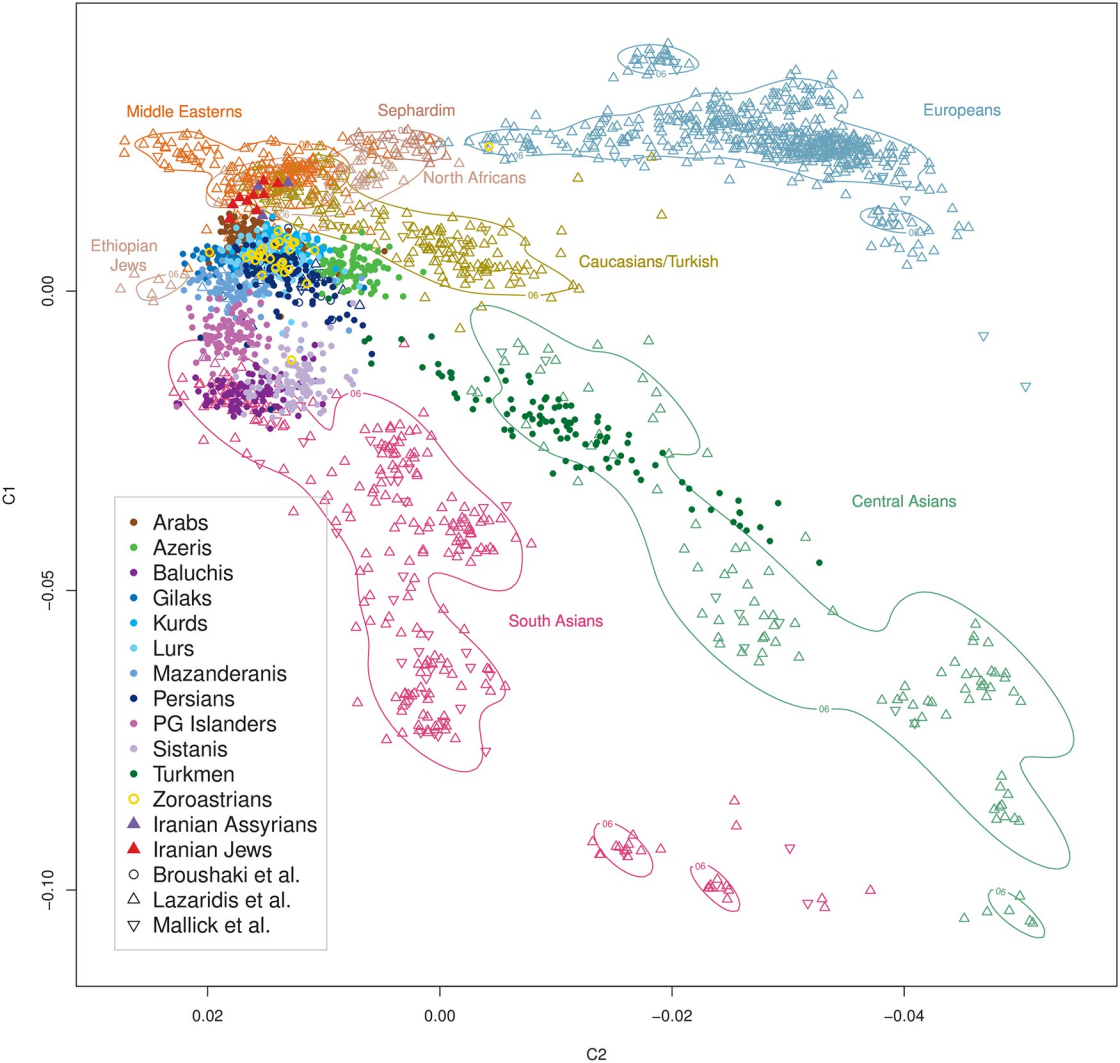
Iranian ethnic groups in a regional context. Relative sample locations with respect to the first two MDS components. Iranian ethnic groups (solid points) in a local context of samples from [2, 6, 44] (open symbols, triangles and 90% density limits).

The 11 included Iranian ethnic groups featured distinct and substantial genetic heterogeneity (Fig 1A). Seven groups (Iranian Arabs, Azeris, Gilaks, Kurds, Mazanderanis, Lurs and Persians) strongly overlapped in their overall autosomal diversity in an MDS analysis (Fig 1B), suggesting the existence of a *Central Iranian Cluster* (CIC), notably also including Iranian Arabs and Azeris. The other four groups (Iranian Baluchis, Persian Gulf (PG) Islanders, Sistanis and Turkmen) presented as strongly admixed populations with contributions by different ancestral populations but always with an orientation towards the CIC, being strikingly different from the CIC and from each other, except for Baluchis and Sistanis who partially overlapped (Fig 1A). On a global scale (Fig 2 including “Old World” populations only; see S2 Fig for all 1000G populations), CIC Iranians closely clustered with Europeans, while Iranian Turkmen showed similar yet distinct degrees of admixture compared to other South Asians. The degree was less pronounced for Baluchis, Sistanis and PG Islanders, with the latter showing a pointed orientation towards Sub-Saharan Africans and a co-localization with numerous Latin American samples. Notably, Iranian Arabs now showed some detachment from the CIC towards Sub-Saharan populations. A local comparison corroborated the distinct genetic diversity of CIC Iranians relative to other geographically close populations [2, 6, 44] (Fig 3 and S3 Fig). Strikingly, the relative genetic location of the Iranian ethnic groups mirrored their geographic location at the nexus between South and Central Asia and West Asia, Northern Africa and the Caucasus. Iranian Baluchis and Sistanis clustered with or nearby Pakistani and other South Asian populations, whereas Iranian Turkmen located next or atop Central Asian populations, respectively. Iranian Arabs appeared distinct from other Arab populations in West Asia and Northern Africa. Furthermore, Zoroastrian samples [6] located as essential CIC members. These results were closely mirrored by the pairwise fixation index (F_ST_) values (Table 2 and S5 Table). CIC groups showed little differentiation (F_ST_~0.0008–0.0033), whereas non-CIC groups consistently yielded much larger values, most extreme for PG Islanders *vs* Iranian Turkmen (F_ST_ = 0.0110). Still, genetic substructure was much smaller among Iranian groups than in relation to any of the 1000G populations, supporting the view that the CIC groups form a distinct genetic entity, despite internal heterogeneity. European (F_ST_~0.0105–0.0294), South Asians (F_ST_~0.0141–0.0338), but also some Latin American populations (Puerto Ricans: F_ST_~0.0153–0.0228; Colombians: F_ST_~0.0170–0.0261) were closest to Iranians, whereas Sub-Saharan Africans and admixed Afro-Americans (F_ST_~0.0764–0.1424) as well as East Asians (F_ST_ ~ 0.0645–0.1055) showed large degrees of differentiation with Iranians. If not corrected for, the observed degree of population substructure could severely confound population-based genetic association studies in Iran. In the extreme scenario of cases being sampled exclusively from one ethnic group and controls from another, CIC groups would yield moderate, although still problematic, genomic inflation factor (GIF) values (1.17–1.61), whereas non-CIC groups may yield values up to 3.0 (Table 2).

**Table 2:** Assessment of population substructure.

Iranian …	Arabs	Azeris	Baluchis	Gilaks	Kurds	Lurs	Mazanderanis	Persians	PG Islanders	Sistanis	Turkmen
Arabs		1.24	2.65	1.50	1.49	1.43	1.61	1.36	2.30	2.08	2.64
Azeris	0.0017		2.39	1.25	1.28	1.24	1.29	1.17	2.28	1.77	2.03
Baluchis	0.0089	0.0073		2.19	2.54	2.43	2.11	2.16	2.62	1.39	2.74
Gilaks	0.0030	0.0015	0.0074		1.37	1.32	1.56	1.23	2.18	1.72	2.42
Kurds	0.0025	0.0013	0.0084	0.0021		1.31	1.41	1.31	2.43	1.98	2.59
Lurs	0.0022	0.0011	0.0076	0.0018	0.0015		1.32	1.19	2.34	1.88	2.52
Mazanderanis	0.0033	0.0016	0.0064	0.0008	0.0023	0.0018		1.23	2.19	1.61	2.36
Persians	0.0018	0.0008	0.0061	0.0014	0.0016	0.0010	0.0012		2.11	1.60	2.21
PG Islanders	0.0070	0.0067	0.0091	0.0076	0.0076	0.0071	0.0068	0.0059		2.21	3.00
Sistanis	0.0058	0.0041	0.0021	0.0043	0.0053	0.0046	0.0034	0.0032	0.0067		2.13
Turkmen	0.0090	0.0056	0.0097	0.0089	0.0087	0.0081	0.0079	0.0067	0.0110	0.0065	

**Lower-left triangle:** Weir’s F_ST_ for pairs of Iranian ethnic groups and for single groups, respectively; **upper-right triangle:** upper bound for genomic inflation factor (GIF) between pairs of groups (see main text for details).

### Ancestry analysis of Iranian ethnic groups

**Fig 4:**
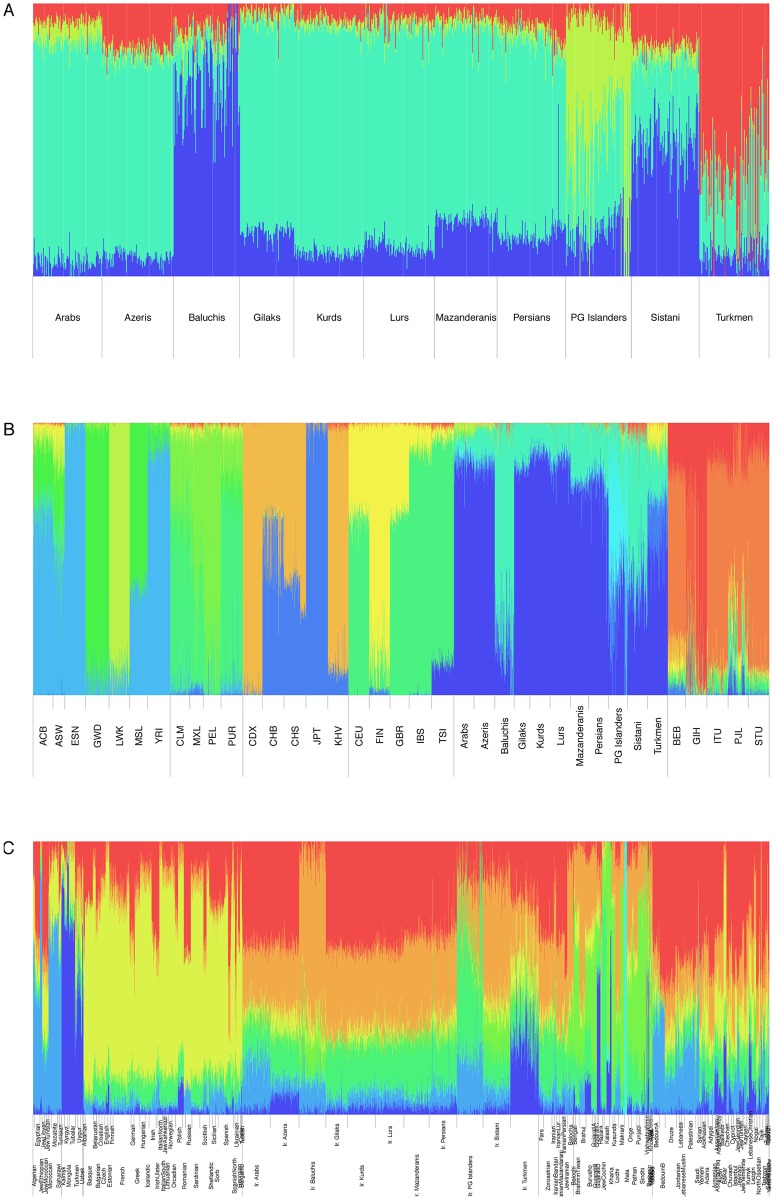
ADMIXTURE inference of Iranian ethnic groups. **(A) Inference in Iranian data set.** Inferred mixture proportions for 1021 Iranian samples from this study for *k* = 4 ancestral populations, yielding a minimal cross-validation (CV) error of 0.544; **(B) Inference in global data set.** Additional inclusion of the global 1000G data set (*k* = 13; CV = 0.499). **(C) Inference in local data set.** Additional inclusion of the local data set (*k* = 8; CV = 0.575).

We further explored the genetic composition and origin of the Iranian ethnic groups. ADMIXTURE [111] analyses corroborated the existence of the postulated CIC and pointed to the existence of a distinct Iranian ancestral component. In the analysis of the 11 Iranian groups alone (best-fit model for *k* = 4), all seven CIC groups featured a single predominant ancestry and slightly varying proportions for the other three ancestral groups, whereas the other four varied in their degree of admixture with different ancestral populations (Fig 4A). Even more strikingly, the global data set analysis (best-fit *k* = 13) yielded three ancestral populations that substantially and almost exclusively contributed to the 11 Iranian groups but were barely seen in the 1000G populations, with one ancestral population shared across all 11 groups (colored blue in Fig 4B) and another one shared by all groups except for PG Islanders which featured a different dominant ancestral population (colored light-green and light-blue in Fig 4B, respectively). A notable exception was the Tuscans (TSI), sharing a substantial proportion of ancestry with Iranians, in particular those from the CIC. A regional comparison corroborated the unique composition of the Iranian ethnic groups (Fig 4C), with Zoroastrian and other Iranian samples showing a concordant picture. Random down-sampling of our Iranian data set to sizes similar those of the reference groups confirmed that this result was not due to our comparatively large sample sizes (S4 Fig). Explicit modeling of 0–15 migration events using TreeMix [112] evidenced the robustness of the close clustering of all Iranian groups, with Europeans always closest to Iranians (S5–S10 Figs). An influx of ancestors from Asian populations to both Turkmen and Finns was consistently inferred, while Iranian Arabs apparently received some African influx. Modelling Iranians as resulting from admixture between pairs of 1000G populations resulted in positive *f*_3_ statistics [113] throughout, thus supporting the primarily autochthonous origin of the CIC groups, except for non-CIC Turkmen that consistently showed negative *f*_3_ values (median -0.0083; range -0.0023 –-0.0096) for any pair of an European and an East Asian population (S6 Table), yielding the strongest evidence for Tuscans admixing Han Chinese or Japanese (*f*_3_ = -0.0093 –-0.0096; Z = -29,2370 –-30,1030). Modelling non-CIC groups as resulting from admixture between a CIC group and a 1000G population yielded a more nuanced picture (S7 Table). While Sistanis consistently appeared to be admixed between CIC and South Asian groups and, less pronouncedly, with Southern Han Chinese, Turkmen revealed components from CIC, African, European, East Asian and, less pronounced, South Asian groups. PG Islanders and also Baluchis comprised a limited African component but no apparent influx from other groups besides the CIC.

### Temporal-spatial relationship of extant Iranians with ancient DNA samples

**Fig 5:**
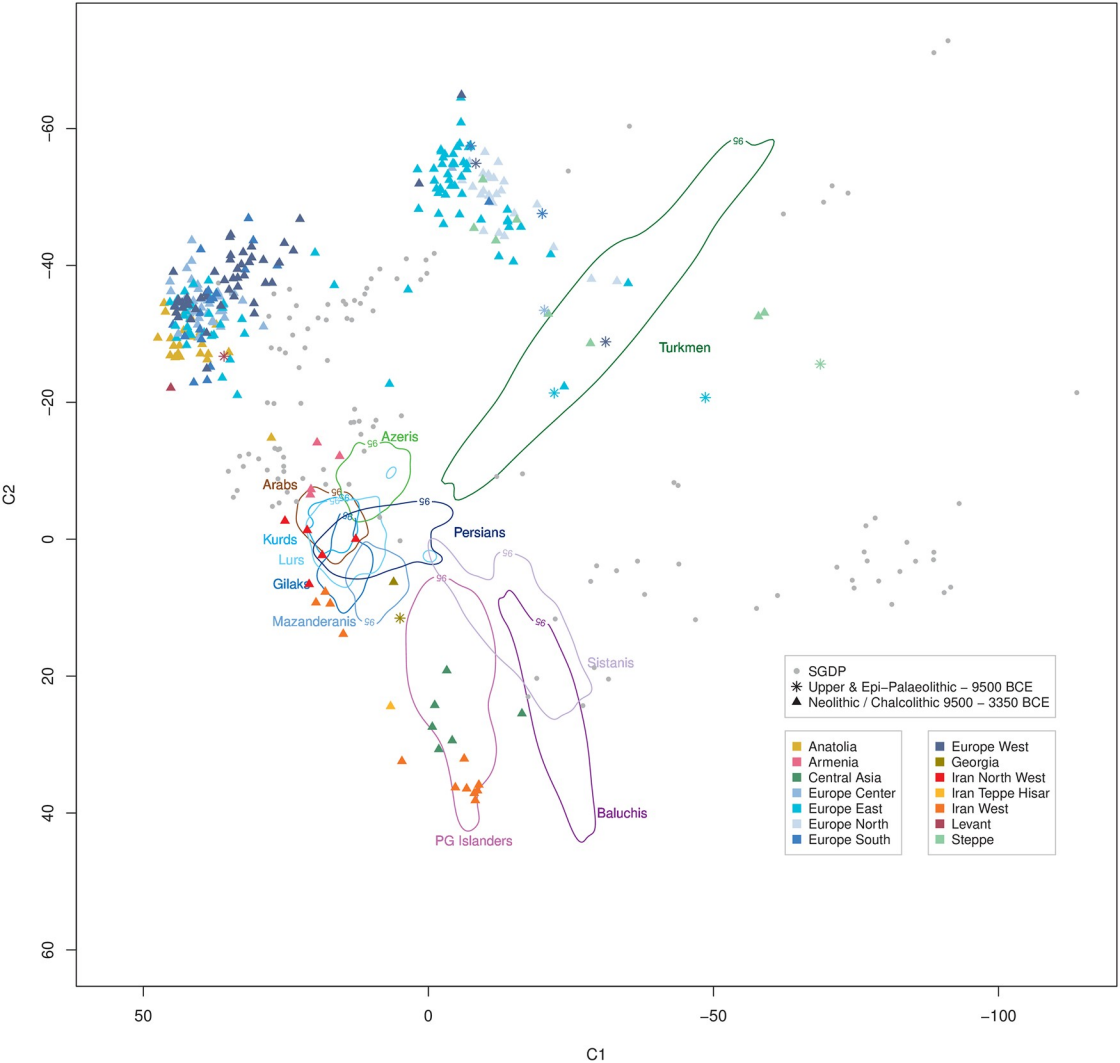
Ancient DNA samples from 45,000 (Upper Palaeolithic)–3350 BCE in the context of extant Iranian ethnic groups. Time-period specific ancient DNA samples (S3 Table) projected onto extant human variation (S18 Fig). The geographic origin of the ancient samples is coded by color.

**Fig 6:**
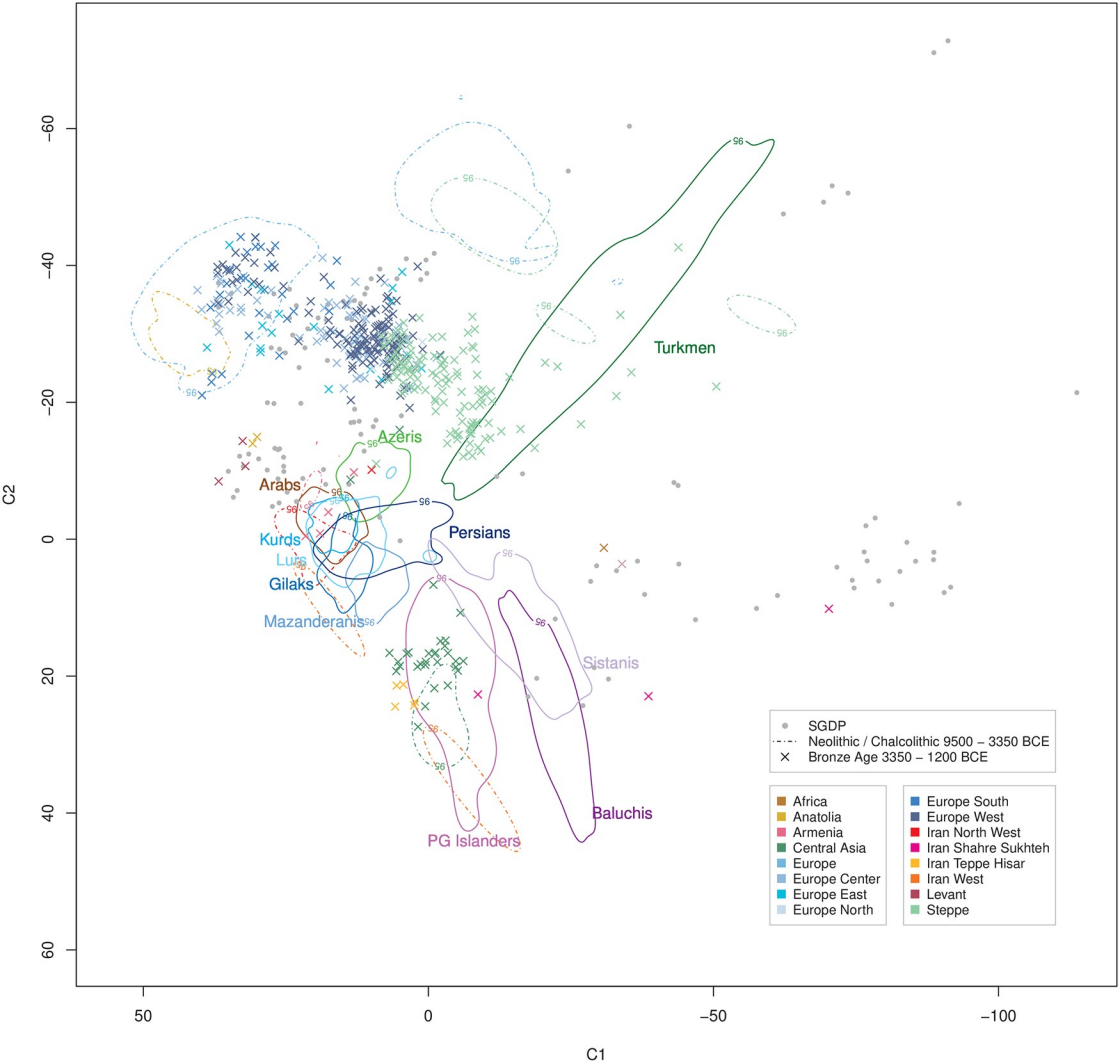
Ancient DNA samples from 3350–1200 BCE in the context of extant Iranian ethnic groups. Time-period specific ancient DNA samples (S3 Table) projected onto extant human variation (S18 Fig). The geographic origin of the ancient samples is coded by color. Previous time strata are indicated by 95% density limits (refer to Fig 5).

**Fig 7:**
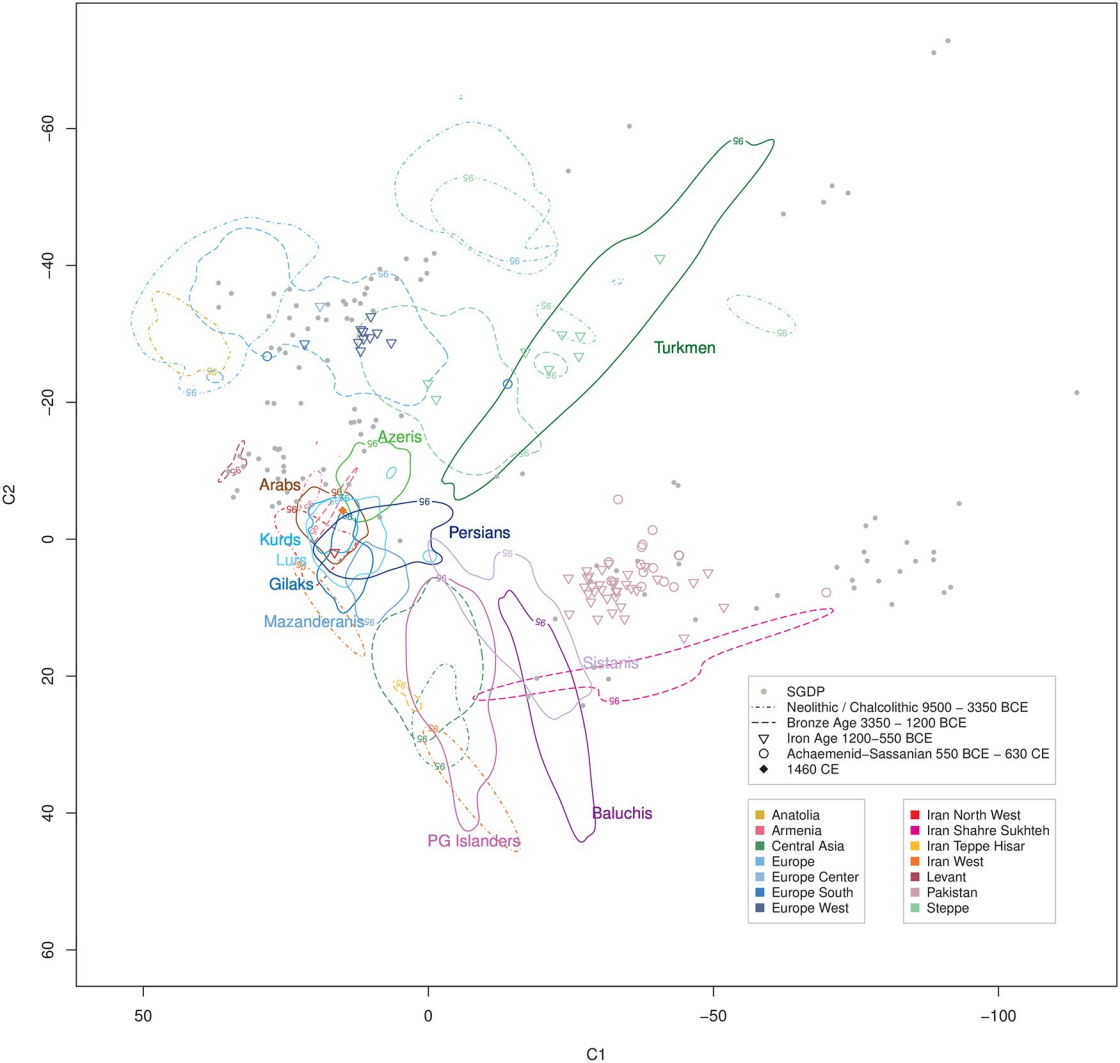
Ancient DNA samples from 1200 BCE–1460 CE in the context of extant Iranian ethnic groups. Time-period specific ancient DNA samples (S3 Table) projected onto extant human variation (S18 Fig). The geographic origin of the ancient samples is coded by color. Previous time strata are indicated by 95% density limits (refer to Figs 5 and 6).

When relating our extant Iranian samples with published ancient DNA (aDNA) samples of different time strata from Iran and beyond to trace temporal-spatial movements of human populations, we did not find indications for substantial migrations into the CIC groups except for Caucasus populations during Neolithic through Bronze Age times (Figs 5–7), with the latter presenting either as a source or as a refuge, i.e. a migration target. In particular, contributions by Steppe people were apparently very limited and restricted to the Bronze Age or briefly before (Fig 6). Overall, the CIC groups appeared to have experienced a largely autochthonous development over at least the past 5,000 years. Remarkably, Early Neolithic Iranian samples [6, 107] from Western Iran and Tappeh Hesar co-localized with the more remotely located extant PG Islanders (Fig 5), whereas later Bronze Age samples from Tappeh Hesar showed a trend towards the CIC (Fig 6), possibly indicating ongoing admixture between these groups. Of note, Central Asian aDNA samples from the Neolithic and the Bronze Age also co-localized with PG Islanders and showed a similar trend (Figs 5 and 6). Sistani samples most distant from the CIC clustered close to Iron Age Pakistani samples (Fig 7) and may have undergone a similar admixture with CIC groups, however, a lack of samples from the past millennia renders this an open question.

### Evidence for several events of language adoption

Languages spoken by the 11 Iranian ethnic groups fell into three different families, namely Afro-Asiatic (Semitic; Arabs), Altaic (Turkic; Turkmen, Azeris) and Indo-European (IE; all others). This linguistic diversity was only partially mirrored by genetic proximity, with Turkic-speaking Iranian Azeris and Semitic-speaking Iranian Arabs closely genetically resembling IE speakers from the CIC, whereas IE-speaking Baluchis, PG Islanders and Sistanis appeared genetically detached from the other IE-speaking groups. After re-classifying our local data set with respect to language family (S2 Table), a general trend of closer genetic proximity, as assessed by a principal-components analysis, for speakers of a language from the same family became obvious (S11A Fig). However, IE speakers fell apart into broadly two distinct groups (corresponding to the European and Indo-Iranian subbranches), while Altaic language speakers comprised widely spread genetic diversity. An approximate autocorrelation analysis based on genetic distance in the first two principal components confirmed a strong localized positive correlation between genetic proximity and spoken language family (S11B Fig).

### Different levels of consanguinity in Iranian ethnic groups

Iran’s ethnic groups strongly differed in their levels of consanguinity. Iranian Arabs, Baluchis and Sistanis showed very high inbreeding coefficient values (F_I_ ~ 0.0122–0.0132), exceeding those of the most consanguineous 1000G population (STU). Iranian Gilaks (F_I_ = 0.0001) and Kurds (F_I_ = 0.0010) showed almost no consanguinity, whereas the other groups showed considerably elevated consanguinity (F_I_ ~ 0.0024–0.069) in comparison to the 1000G populations (S12A Fig and Table 3). Of note, consanguinity varied widely within each group, with 50% of individuals showing F_I_ values below 0.0051 (Iranian Arabs), 0.0042 (Iranian Sistanis) and 0.0036 (Iranian Baluchis), respectively, and virtually equal to zero in the remaining groups. Cumulative lengths of IBDseq-inferred autozygous regions and of PLINK-defined runs of homozygosity (ROHs) closely mirrored the distribution of inbreeding values (S12B and S12C Fig). Likelihood-based ROH definition and subsequent length classification by GARLIC (S12D–S12F Fig) revealed substantial amounts of ancestral class-A cumulative ROH length in virtually all Iranian ethnic groups and 1000G populations but also generally much shorter recent class-C cumulative ROH length. Iranian Arabs, Baluchis and Sistanis most prominently deviated from this trend, while most other Iranian groups showed still elevated values, indicating ongoing consanguinity through the past millennia.

**Table 3:** Comparative consanguinity assessment.

	F_I_	Runs of homozygosity (PLINK)		Autozygous segments (IBDseg)		Class C segments (GARLIC)	
Iranian …		Number	Cumulative [Mb]	Number	Cumulative [Mb]	Number	Cumulative [Mb]
**Arabs**	0.0122±0.0192	43.9±9.7	127.7±96.6	21.6±8.8	91.2±102.6	2.9±3.7	54.2±75.9
**Azeris**	0.0025±0.0127	40.6±7.7	82.5±67.8	17.1±6.6	40.5±70.2	1.2±2.7	21.8±53.9
**Baluchis**	0.0123±0.0213	51.1±12.2	156.1±118.8	20.9±11.2	114.4±122.0	2.8±3.8	59.0±86.6
**Gilaks**	0.0001±0.0104	43.1±12.3	84.0±73.2	11.0±9.2	34.8±70.2	0.6±1.7	11.3±30.6
**Kurds**	0.0010±0.0100	42.5±7.1	82.3±52.7	15.2±5.3	36.8±55.6	1.1±2.3	19.2±41.6
**Lurs**	0.0048±0.0137	43.4±9.5	100.2±81.3	18.0±8.1	57.6±85.8	1.4±2.8	27.0±55.4
**Mazanderanis**	0.0037±0.0110	44.5±9.0	95.1±60.4	17.1±8.1	49.9±62.3	0.5±1.0	13.7±26.0
**Persians**	0.0057±0.0149	42.9±9.6	97.9±74.2	18.3±9.0	56.1±78.5	0.9±1.8	21.0±40.4
**PG Islanders**	0.0024±0.0114	42.1±9.2	97.9±74.2	12.2±7.7	60.3±74.0	1.9±2.8	31.2±50.6
**Sistanis**	0.0132±0.0202	48.1±12.3	147.9±111.5	23.1±11.3	110.0±119.1	2.6±3.4	53.4±71.4
**Turkmen**	0.0069±0.0167	38.7±8.5	99.0±82.9	17.9±8.4	62.0±85.1	1.4±2.4	32.1±57.9

Given are mean±standard deviation for selected indicators of autozygosity in the Iranian ethnic groups.

Akin to previously studied populations, the genomic distribution of PLINK-defined ROHs followed a highly non-uniform pattern that was highly concordant across all groups (S13A Fig) and similar to that obtained for the non-African 1000G populations (S14 Fig; analysis performed on the markers present in the merged data set), with a number of ROHs reaching substantial frequencies in the Iranian population (S8 Table). CNVs, as defined by the Axiom Analysis Suite v4.0 software, were predominantly detected in Iranian Gilaks, Mazanderanis and Sistanis (S15 Fig) and also comprised a highly non-uniform genomic distribution that showed virtually no systematic overlap with ROHs (S13B and S13C Fig), resulting in a number of high-frequency CNV regions (“CNV islands”; S9 Table) in healthy individuals.

### Differences in allele frequencies across Iranian ethnic groups

The observed genetic diversity and partially different ancestry was also evident in the frequency differences for numerous trait-related or predisposing alleles in the Iranian ethnic groups (S10 Table). In general, CIC groups tended to have very similar allele frequencies that were nevertheless often markedly different from those of Europeans, while Iranian Baluchis and Sistanis showed a tendency towards South Asians, although these trends were not present at all markers. A notable exception was lactase persistence-causing marker allele rs4988235-T whose frequency in Iranian Baluchis (22%) was much higher than in any of the other Iranian groups, raising the prospect of convergent evolution [114]. However, we did not find evidence for a selective sweep based on Tajima’s D (S16 Fig) nor when using the integrated haplotype score (iHS) approach [115] (S17 Fig). Although rs4988235 showed a substantial absolute score in Baluchis (|iHS| = 2.42), this value was not significant (two-sided p>0.05) and we also did not observe a clear clustering of SNPs with extreme values as a possible indication for positive selection [116].

## Discussion

Our study, based on genome-wide data from a stratified ethnic-group sampling and also including groups previously not well covered, such as Iranian Gilaks, Kurds, Mazanderanis and Sistanis, revealed the distinct and rich genetic diversity of the Iranian population, corroborating previous reports based on uniparental markers. The majority of Iran’s ethnic groups comprise largely overlapping genetic autosomal diversity, implicating a shared and largely autochthonous ancestry, designated as the *Central Iranian Cluster* (CIC). Notably, the CIC also includes Iranian Arabs and Azeris (Fig 1) as well as the religious group of Zoroastrians (Fig 3), being consistent with the suggestion that Zoroastrians have lived in the area of present-day Iran for millennia and had formed an early group of Indo-European speakers. Still, the CIC comprised substantial internal structure, with pairwise F_ST_ values up to an order of magnitude higher than those for more homogeneous populations of similar population size, such as Germany [117], but below the level of substructure reported for Europe, Central Asia, the Near East or Southeast Asia as a whole [45] and much lower than for neighboring Armenia in the Caucasus [118]. Iranian Baluchis, Sistanis, Turkmen and Persian Gulf Islanders showed strong admixture, with the CIC (or its ancestral population) consistently contributing to all of them and contributions from different respective ‘opposite’ ancestral populations, evidencing CIC’s strong impact on human demography in this world region. Since substantial proportions of the Iranian population belong to non-Persian ethnic groups or are admixed, more precise reference to the particular ethnic groups appears mandatory when conducting future genetic studies.

In comparison with global and local reference data, the CIC represents a distinct entity comprising an autochthonous genetic component, clustering closely with geographically adjacent populations and assuming a location in the ‘genetic map’ that corresponds to its geographic location at the nexus between South, Central and West Asia, Northern Africa and the Caucasus. This observation is consistent with limited gene flow reported in previous uniparental marker-based studies and adding a further example on the correspondence between genetic diversity and geographic location, such as Europe [73, 119], explicable by genetic drift as well as admixture. The largely autochthonous development of CIC groups, consistent with an early branching from the Eurasian population before the Neolithic [6], is further corroborated by the distinctiveness of these groups in comparison to different time strata represented by aDNA samples, indicating a genetic continuity for at least several past millennia and eventually mirrored by Zoroastrian genomic diversity. Both, Early Neolithic farmers from West Iran and people from the Steppe appear to have made very limited contributions to CIC groups. In turn, the ‘African’ component shared between PG Islanders and some Sub-Saharan populations likely predates the beginning of the Neolithic and, thus, renders PG Islanders as an early autochthonous group that subsequently became strongly admixed with CIC groups. Notably, Iranian Arabs appear to be slightly genetically detached from other Arab populations in West Asia and Northern Africa. The small ancestry component shared between the CIC and Tuscans may mirror early migrations from the Near East although this requires further investigation.

Correlating genetic affinity with spoken language yielded evidence for a number of language adoption cases in Iran. CIC’s distinct and autochthonous genetic variation indicates that Indo-European (IE) language(s) were likely adopted by some ancient population in Iran several millennia ago, although it remains unclear if this was driven by previously suggested aggressive warrior-bands migration [120] given the lack of Y-chromosomal data in our study. The observed close genetic proximity, based on genome-wide data, of Turkic-speaking Iranian Azeris as well as of Semitic-speaking Iranian Arabs to IE-speaking groups within the CIC, confirms previous reports on Semitic-speaking groups in Iran [58] and Turkic-speaking Azerbaijanis [91, 121–123]. Given their genetic vicinity to other Arab and Caucasian populations, respectively, this is well explained by admixture between some overwhelmingly contributing ancestral IE population(s) and a minor genetic contributor whose language was adopted in the course of past entries. Finally, the spread of IE-speaking Iranian Baluchis, Sistanis and PG Islanders from the other IE-speaking CIC groups is explicable by repeated admixture of some IE-speaking ancestral population(s) with ancient South or West Asian populations, such as Early Neolithic West Iranians, respectively, while retaining their language, causing its adoption by the admixed offspring.

The heterogeneous levels of substantial population substructure as well as of elevated consanguinity in the Iranian population have profound implications for future human genetic studies. They corroborate previous reports on different predisposing variant frequencies across Iran (e.g. [36, 37]) and emphasize the need for an ethnicity-aware approach when performing human genetic studies or genetic counseling in Iran. Population-based association studies should focus on CIC groups to minimize biasing effects due to population stratification, applying to common single-marker analysis but in particular to rare-variant collapsing tests where regional and ethnic group-specificity is to be expected due to the average young age of these variants. Given the genetic diversity even within the CIC, ancestry correction appears mandatory while sample inclusion from the highly admixed groups may increase the risk of biased results. The observed elevated consanguinity in some ethnic groups is in line with previous reports on Iran and other West Asian populations, indicating past and ongoing consanguineous marriage practice and also possibly explaining reported differences between Iranian provinces and residential areas. Family-based linkage or homozygosity-mapping studies should preferentially target groups featuring increased consanguinity levels, namely Iranian Arabs, Baluchis and Sistanis, to increase power especially for studying autosomal-recessive diseases. When studying runs of homozygosity and copy-number variants in diseased individuals, for example in whole-exome and whole-genome sequencing studies, the frequent occurrence of such features in healthy individuals, as shown in this work, requires caution in the interpretation of these features.

In summary, Iranians feature distinct genetic variability, resulting from long-standing genetic continuity, as well as substantial genetic heterogeneity and can, thus, not be treated as a single homogeneous entity. Future human genetic studies have to consider ethnic affiliations for sampling and analyses and should expect widespread admixture in both extant and ancient samples. The observed concordance between genetic diversity and geographic location and examples of lineage break up between language and genetic proximity are consistent with the archeological and historical evidence on Iran as occupying a stretch of land that has seen multiple migration and admixture events in the past millennia. By providing genome-wide population data for Western Asia, thereby filling a lack that has characterized this region for over a decade despite its known diversity and prominent place in human history, we hope to encourage future population genetic, evolutionary and medical studies in Iran and beyond.

## Material and methods

### Ethics statement

This study has been approved by the Research Ethics Committee of the University of Social Welfare and Rehabilitation Sciences (USWR), Tehran, Iran (approval number IR.USWR.REC.1395.376). Prior to gathering information on sex, ethnicity, demographic and health status, we obtained written informed consent from each individual, according to the guidelines of the Research Ethics Committee, University of Social Welfare and Rehabilitation Sciences (USWR), Tehran, Iran.

### Iranian study samples

We included 1069 healthy unrelated individuals from 11 major Iranian ethnic groups, including 800 from the Iranome project [124] as well as 269 additionally sampled individuals in the study, comprising Iranian Arabs, Azeris, Baluchis, Kurds, Lurs, Gilaks, Mazanderanis, Sistanis, Persians, Turkmen and Persian Gulf Islanders living in Iran (Table 1). Prior to gathering information on sex, ethnicity, demographic and health status, we obtained written informed consent from each individual, according to the guidelines of the Research Ethics Committee, University of Social Welfare and Rehabilitation Sciences (USWR), Tehran, Iran. Individuals were required to have the same ethnic background for at least two generations. The majority of individuals were more than 40 years old at the time of recruitment, lowering the risk of manifesting genetic disorders in later life. All subjects were re-examined by a clinician. This study has been approved by the Research Ethics Committee of USWR, Tehran, Iran. Language family assignment was obtained from Glottolog 3.2 (http://glottolog.org/).

### Sample processing, genotyping and data quality control

Venous blood was taken from individuals. DNA extraction from blood samples was done using the salting out method [125]. Samples were genotyped using the Axiom Precision Medicine Research Array (PMRA) by Life Technologies, comprising about 903,000 markers. Samples were randomly assigned to genotyping array probes without regard to ethnic affiliation in order to avoid batch effects. Life Technologies’ AxiomAnalysisSuite v2.0.0.35 was used for evaluating and genotyping CEL files. After removing low quality samples (quality < 97), genotypes of 1058 samples were assigned using the Axiom_PMRA.na35.annot.db annotation file. Further quality control was performed on those 1058 samples using PLINK [126] v1.9 and R v3.5.1 [127]. Variants were required to have call-rates ≥95% and deviations from Hardy-Weinberg equilibrium with p>10^−5^. Samples were required to have a call-rate of ≥97%, to not show excessive hetero- as well as homozygosity (<5 sd). Cryptic relatives (mean identity-by-descent [IBD] sharing π>0.4) were detected using PLINK’s—genome option and 20 samples were excluded from the study for representing parent-child pairs, sib pairs or identical individuals. After quality control, the cleaned data set comprised 1021 samples (Table 1) comprising genotypes for 829,779 autosomal markers. The overwhelming majority of sample pairs within an ethnic group (typically ~99% or more) were unrelated or only distantly related (π<0.04125), with only few pairs showing elevated IBD sharing (S11 Table). For some analyses, we additionally considered only markers with common alleles (minor allele frequency ≥5%; 311,262 markers), only markers in no strong linkage disequilibrium (LD; r^2^≤0.5, 500kb window size, 25 SNPs step size) by using PLINK’s—indep-pairwise option (475,665 markers), or both (203,495 markers).

### Human reference data sets

In order to put the Iranian samples in a global as well as local context, we merged our cleaned data set with those of publicly available reference data sets, using only markers that were present in each of the datasets being merged. For a global comparison, we used 2492 unrelated samples assigned to 26 populations from the 1000 Genomes Project [41–43] (“1000G”; accessed May 2017). For a more localized comparison, we used samples from three different curated data sets, namely 120 samples from the Simons Genetic Diversity Panel (SGDP) [44], 1345 samples from Lazaridis et al. [2], partially including previously published samples, and 45 samples from Broushaki et al [6]. Notably, these reference data also included samples from a wide variety of ethnicities, such as Semitic groups (e.g. Arabs, Assyrians, Jews), Caucasian groups (e.g. Armenians, Georgians, Circassians), Zoroastrians and many others. We further grouped these samples for their corresponding geographic region (S1 Table) and language family (S2 Table). Only markers with genotypes in both the Iranian and the respective reference data set(s) were included in the analysis and underwent additional quality control using the same thresholds as before. Again, for some analyses, markers in strong LD or with infrequent alleles were removed. After QC, the ‘*global data set’* (merger with 1000G) included 782,127 markers, while 232,138 common markers remained after additional LD pruning and frequency filtering. The ‘*local data set’* (alternative merger with the other three reference data sets) included 59,837 markers in total and 43,198 common, LD-pruned markers, respectively. A growing number of human aDNA samples from Iran and beyond have been published. We compiled 798 aDNA samples from 21 different publications and one pre-print [2, 6, 81, 92–110] (S3 and S4 Tables) for spatial-temporal analysis.

### Population differentiation and admixture assessment

We applied multidimensional scaling (MDS) analysis based on identity-by-state (IBS) allele sharing to the LD-pruned data sets using PLINK’s—mds-plot implementation. PCA analysis was independently performed for each of the considered, possibly merged, data sets, except for the aDNA samples which were projected onto the components obtained from the merged data set of our 1021 extant Iranians and 118 SGDP samples geographically co-localizing with the aDNA samples (S4 Table; S18 Fig). This data set underwent quality control, LD pruning, and frequency filtering using the same thresholds as before. We generated PCs of reference samples running TRACE from LASER [128] v2.04 in PCA mode (-pca 1) using default parameters. Then we projected each aDNA sample independently onto the reference PCA using TRACE [128] v1.03 with default parameters. The number of markers each aDNA sample shared with reference PCA ranged from 30,000 to 80,000. PLINK’s—fst option was used to estimate Weir & Cockerham’s F_ST_ fixation index [129]. For an approximate assessment of the upper limit of the impact of population substructure on genetic association studies in the Iranian population, we deliberately assigned, for each pair of ethnic groups, case status to all samples from one group and control status to all from the other and subsequently calculated the genomic inflation factor [130] (GIF), where values of 1.0 correspond to no inflation, by using PLINK’s—adjust option. For exploratory admixture and migration analysis, we ran ADMIXTURE [111] v1.3.0 in parallel for K = 2, …, 20 using random seeds and TreeMix [112] v1.13 through the Treemix_bootstrap.sh script of the BITE R package [131] v1.1.0004 allowing for 0, 1, 2, 5, 10 and 15 migration events to be replicated 100 times and made consensus trees based on replications using PHYLIP [132] v3.697. The final tree was then plotted using treemix.bootstrap from BITE. The qp3Pop program of ADMIXTOOLS v5.0 package with default parameters was used to calculate f_3_ statistics [113].

### Language autocorrelation analysis

Autocorrelation analysis for language family with respect to genetic distance based on the Euclidian distance in the first two principal components and Moran’s I [133], obtained from running the TRACE software from LASER v2.0 [128] on the local data set, was performed using the Moran.I function from R package ape v5.1 [134]. To this end, language families were assigned numeric class values (1,…,8). To avoid spurious effects due to this numeric (instead of categorical) coding, which would imply order and distance between classes, we performed 100 random permutations of the assigned values, thereby destroying potential biases introduced by arbitrary numbering, and report the respective distributional statistics.

### Selection analysis

Tajima’s D was estimated with VCFtools v0.1.13 (https://vcftools.github.io/man_latest.html) using the—TajimaD option with a window size of 100 kb and the—from-bp/—to-bp commands for a sliding window analysis. We also performed an integrated Haplotype Score (iHS) analysis [115] of the *LCT* region on chromosome 2. To this end, haplotypes were estimated using ShapeIt v2.r790 [135] based on the 1,000 Genomes Phase 1 haplotype reference panel and genetic map of chromosome 2 (downloaded from: https://mathgen.stats.ox.ac.uk/impute/data_download_1000G_phase1_integrated.html). A total of 34,746 SNPs on chromosome 2 coincided between our data set and the reference panel. The iHS scan was performed using R package *rehh* v2.0.2 [136, 137] (downloaded from: https://cran.r-project.org/web/packages/rehh/index.html).

### Autozygosity and copy-number variant assessment

Inbreeding coefficients (F_I_) were estimated using PLINK’s—ibc option (‘Fhat3’; [138]) based on LD-pruned autosomal markers and separately for each ethnic group. Furthermore, we defined runs of homozygosity (ROHs) using PLINK v1.9 (LD-pruned autosomal markers; ethnic groups combined) and GARLIC [31, 139] v1.1.4 (autosomal markers; separately for each ethnic group) and autozygous genomic regions using IBDseq [32] (LD-pruned autosomal markers; separately for each ethnic group), using default options and applying them to separate data subsets containing only a single population or ethnic group, respectively. We used the Axiom Analysis Suite (AxAS v4.0) with default options in order to detect copy-number variants (CNVs). We divided the set of 1021 Iranian samples into 5 groups where each group comprised similar proportions of males and females from the 11 ethnic groups. Samples in each group were used to construct a reference for CNV detection, subsequently running the CNV detection for the same groups. CNVs were required to comprise at least 25 and 50 markers for homozygous and heterozygous variants, respectively.

### Statistical analysis

All statistical analyses were performed and graphs were created using R with in-house scripts, unless noted otherwise. Two-dimensional kernel density estimates were obtained using the Hpi and kde functions from the ks package v1.11.3 [140] for R. Map data were obtained from GADM v2.8 (November 2015; www.gadm.org) and maps were plotted using functions in the sp package v1.3–1 [141, 142] for R. Bar plots were created using functions in ggplot2 v3.0.0 [143] for R.

## Supporting information

S1 Appendix Description of the historic, ethnic and linguistic background of Iran. (PDF)

S1 Fig Map of Iran, its provinces and surrounding countries. Regions with predominance of a particular ethnic group are designated by color. Adapted, modified and simplified from [8] and http://legacy.lib.utexas.edu/maps/iran.html (Perry-Castañeda Library Map Collection, The University of Texas at Austin, USA; file iran_country_profile_2009.jpg).
(TIF)

S2 Fig Iranian ethnic groups in a global context. Relative sample locations with respect to the first two MDS components. Iranian ethnic groups in a global context (global 1000G data set, with 90% density limits); *inlet*: zoomed view of the CIC and adjacent European populations.
(TIF)

S3 Fig Regional location of extant Iranian ethnic groups. **A.** Regional context. Relative sample locations in the first two MDS components based on the samples from this study and the local reference data set, including 90% density limits. European samples were omitted for clarity. **B.** Zoomed view of (A) to the CIC and adjacent groups.
(TIF)

S4 Fig ADMIXTURE inference of Iranian ethnic groups in the local data set. Inference was based on the local data set, but with the Iranian samples randomly down-sampled to assess a potentially biasing effect of the comparatively larger sample sizes compared to the other populations from the region. For each of the seven CIC ethnic group, 5 samples were randomly drawn, whereas the other four non-CIC groups contributed 20 random samples each. Given are the solutions providing the smallest errors. **A:** *k* = 7, CV = 0.57390; **B:** *k* = 8, CV = 0.57393.
(TIF)

S5 Fig TreeMix-based admixture inference of the global data set assuming no migration event. Nodes are colored by bootstrapping support, edges by weight of migration.
(TIF)

S6 Fig TreeMix-based admixture inference of the global data set assuming a single migration event. Nodes are colored by bootstrapping support, edges by weight of migration.
(TIF)

S7 Fig TreeMix-based admixture inference of the global data set assuming two migration events. Nodes are colored by bootstrapping support, edges by weight of migration.
(TIF)

S8 Fig TreeMix-based admixture inference of the global data set assuming five migration events. Nodes are colored by bootstrapping support, edges by weight of migration.
(TIF)

S9 Fig TreeMix-based admixture inference of the global data set assuming 10 migration events. Nodes are colored by bootstrapping support, edges by weight of migration.
(TIF)

S10 Fig TreeMix-based admixture inference of the global data set assuming 15 migration events. Nodes are colored by bootstrapping support, edges by weight of migration.
(TIF)

S11 Fig Language family *vs* genetic similarity in the local data set. **A.** PCA plot of the local data set, colored by spoken language family. See S2 Table for language assignments. **B.** Autocorrelation analysis. Distance between pairs of individuals defined by the Euclidean distance with respect to the first two principal components.
(TIF)

S12 Fig Comparative individual consanguinity and homozygosity assessment in the global data set. **A.** Inbreeding coefficient F_I_; **B.** IBDseq-defined autozygous regions (HBD). Total genomic autozygous sequence [Mb]; **C.** PLINK-defined runs of homozygosity (ROHs). Total genomic sequence [Mb] of ROHs; **D-F.** GARLIC-defined ROHs. Total genomic sequence [Mb] located in class A short and likely ancient ROHs (D), class B intermediate-length ROHs (E) and class C long and likely recent ROHs (F), respectively.
(TIF)

S13 Fig Highly concordant genomic distribution of selected features across Iranian ethnic groups. **A.** Density of PLINK-defined runs of homozygosity (ROHs). Sample proportion per group featuring an ROH at a given genomic location. **B.** Density of copy-number variation (CNV). Sample proportion per group featuring any of four CNV types at a given genomic location. **C.** CNV type density in the Iranian population. Total: cumulative portion over all four CNV types; 0: loss of both copies; 1: loss of one copy; 3: gain of one copy; 4: gain of two copies.
(TIF)

S14 Fig Density of PLINK-defined runs of homozygosity (ROHs) in Iranian ethnic group and the 1000 Genomes populations. Sample proportion per group featuring an ROH at a given genomic location.
(TIF)

S15 Fig Number and extent of CNVs detected in Iranian ethnic groups. **A.** Number of detected CNVs per group. **B.** Cumulative sequence length [kb], i.e. total length of genomic sequence included in CNVs. **C.** Average sequence length per CNV [kb].
(TIF)

S16 Fig Tajima’s D in Iranian ethnic groups. **A.** Autosome-wide distribution density of Tajima’s D (100 kb window size), separately for each Iranian ethnic group. **B.** Local Tajima’s D values around rs4988235 (100 kb window size; 10 kb shift) in Baluchis. Red line: rs4988235; orange line: rs16832011.
(TIF)

S17 Fig Selection analysis based on the iHS around the *LCT* region in Baluchis. Shown are extreme absolute iHS values on chromosome 2 around the *LCT* gene region. **Black dots:** SNPs with |iHS|>2; **green dots:** SNPs with |iHS|>2.63, there representing the top 1% of SNPs on chromosome 2 with the largest absolute iHS values (in line with [115]); **red dot:** lactase persistence SNP rs4988235 (iHS = -2.51); **grey dashed lines:** borders of the *LCT* (136.54–136.59 Mb) and *MCM6* (136.59–136.63 Mb) gene regions according to GRCh37/hg19 (www.genecards.org; [116]).
(TIF)

S18 Fig Background for projecting ancient DNA samples in relation to extant Iranian ethnic groups. First two MDS components defined by the Iranian groups from this study (indicated by 95% density limits) and selected SGDP [44] samples from the region (S4 Table; dots).
(TIF)

S1 Table Assignment of reference samples to population supergroups in the local data set. Samples from selected ethnic groups or sampling sites from [2, 44] were assembled to supergroups for better visualization.
(DOCX)

S2 Table Language family assignment of ethnic groups in the local data set. *: Present in this study’s data set. Assignment has been based in the following sources: https://www.ethnologue.com/browse/countries; http://glottolog.org/; http://wals.info/languoid.
(DOCX)

S3 Table Compiled ancient DNA (aDNA) samples and their sources. 798 aDNA samples were obtained from 21 different publications and 1 pre- print [2, 6, 81, 92–110].
(XLSX)

S4 Table Assignment of SGDP reference samples to population supergroups in the aDNA plot. (DOCX)

S5 Table Weir’s F_ST_ for pairs of an Iranian ethnic group and a 1000 Genomes population. (DOCX)

S6 Table *f*_3_ statistics for Iranian ethnic groups resulting from admixture between 1000 Genomes populations. Each sheet refers to an Iranian ethnic group being modeled as resulting from the admixture between the row and column the 1000 Genomes populations. Lower-left triangle: *f3* statistic values; upper-right triangle: corresponding Z values. Negative *f3* values are shaded.
(XLSX)

S7 Table *f*_3_ statistics for non-CIC Iranian ethnic groups resulting from admixture between a CIC group and a 1000 Genomes population. Left table: *f3* statistic values; right table: corresponding Z values. Negative *f3* values are shaded.
(XLSX)

S8 Table Genomic regions comprising high ROH frequency in the Iranian ethnic groups. Sheets list the location of regions where ROHs occur with a frequency of at least 0.95, 0.90, etc., respectively, in the Iranian population.
(XLSX)

S9 Table Genomic regions comprising high CNV frequency in the Iranian ethnic groups. Sheets list the location of regions where CNVs occur with a frequency of at least 0.40, 0.30, etc., respectively, in the Iranian population.
(XLSX)

S10 Table Allele frequency estimates for selected SNPs. Estimates are given separately for the Iranian ethnic groups and the 1000 Genomes populations.
(XLSX)

S11 Table Pairwise identity-by-descent (IBD) sharing within Iranian ethnic groups. (DOCX)
