# Supplementary material for: Distinct genetic variation and heterogeneity of the Iranian population
Source: PLoS Genet. 2019 Sep 24;15(9):e1008385. doi: 10.1371/journal.pgen.1008385 (PMC6759149; doi:10.1371/journal.pgen.1008385)
Supplement: S1 Appendix — (PDF) [file pgen.1008385.s001.pdf]

Description of the historic, ethnic and linguistic background of Iran

Historic sketch of Iran.

The modern state Iran comprises the Iranian highlands and central deserts as well as the majestic mountain ranges of Alborz and Zagros. Albeit vast, the modern country is much smaller than Iranian territory was throughout parts of her history. Rugged terrain, an uninhabitable central desert and topographical constraints on communication routes have shaped territorial cultural and political formations over time that play out in today's (self-) defined ethnic formations. Two antagonistic principles can be outlined that characterize the history of Iran: on the one side an Iranian (Persian) political and cultural hegemony over a vast territory ensuring infrastructure like roads that promoted long distance contacts. Since the appearance of nomadic tribes in the wake of Turkic immigrations since the 11th century, long distance mobility remains a major characteristic of Iran. And on the other side, cultural decisions maintaining linguistic or religious group identities through rules like endogamous marriage.

In the early 2nd millennium BCE, Iran was inhabited by populations of diverse linguistic background. Elamites lived in the south Iranian Zagros and its adjacent coastal plains, undocumented language communities thrived in the oases of eastern Iran and the Hilmand Basin, and for many other regions we have no evidence for the linguistic affiliation of their populations. At this time a first influx of speakers of a proto-Indo-Aryan language is documented with Hurrian groups in the high valleys of Northwestern Iran and extending into the plains of northern Syria. The mosaic changed in the 2nd half of the 2nd millennium BCE when groups speaking western Indo-European (IE)/Indo-Iranian (IIr) languages began to appear in the north of Iran. Their origin can only be hypothetically reconstructed as the archaeological cultures did not leave written records and cannot be directly linked to any language group. Linguists use loanwords exchanged between IIr and Uralic languages to reconstruct a most likely scenario of origin in the South Russian western steppe belt between Ukraine and Kazakhstan ([1], p. 427-428). This roughly corresponds to the region where in the 2nd millennium BCE the archaeological culture labelled "Andronovo" flourished [2]. As, however, no Andronovo material is found along the alleged migration route of IIr into Iran ([3], p. 71), this hypothesis cannot be positively confirmed.

In the 1st millennium BCE, Iranian languages were spoken all over southern and western Iran and had largely absorbed their local predecessors like Elamite. One of them, Old Persian, became the hallmark of the Achaemenid Empire (550-330 BCE). Persian language and culture spread over a large territory that incorporated numerous regional cultures and

languages. The imperial project also led to the resettling of numerous people from all over the empire, often craftsmen moved to construction sites to profit from their specific skills [4]. The Achaemenid administration established routes and trade posts facilitating long distance contacts and maintaining a stable Persian cultural koiné, which promoted the spreading of Zoroastrian faith. Interludes such as Alexander's conquest in 330 BCE and the Seleucid rule (312-63 BCE) that was cut short in Iran through the establishing of the Parthian (250 BCE-224 CE), followed by the Sassanian (224-651 CE) dynasties, did not seriously interrupt cultural continuity. With the Muslim conquest of Iran in 651, Sunni Islam started to spread slowly. The Caliphs established military posts and administrators in Iran, thus promoting the immigration of Arabian settlers. In the 11th century began a process of Turkic infiltration into Iran: the first Turkic tribe to appear in Iran from the northeast were the Seljuqs who defeated the Ghaznavid dynasty ruling in East Iran and formed a hybrid Turko-Persian culture. Subsequently Turkic tribes spread rapidly over wide parts of northern Iran. Turkish influence onto Iran grew exponentially in the 13th century in consequence of the disastrous Mongol invasion led by Jengiz Khan against Khorassan (1219-1223) and his grandson Hülegü against the Iranian highlands, and resulted in the death and replacement of parts of the sedentary population. In the Timurid era (1370-1507) began a partial eastwards reflux of Turkic tribes from Syria and Anatolia into Iran. The Safavids (1501-1722), who shifted their capital to Isfahan, introduced Shia Islam as leading religion and drove many Zoroastrians into exile. Nomadic tribes living on the western periphery of the empire like the Baluch were resettled to the Southeast. Tribal deportations became a probate means to suppress rebellions since the reign of Shah Abbas (1588-1629). Afghan tribes conquered Isfahan in 1722, and the next decades saw the division of power between competing regional rulers until the rise of the Qadjars toward the end of the 18th century. The 19th century is regarded as a "peak" of tribal unrest, that the central government countered with tax laws that pressed for sedentarisation ([5], p. 294); serial epidemics of plague and cholera depopulated wide areas ([5], p. 305-6) even before the severe famine in 1870/71 left a minimum of 20% of the population dead [6]. Qadjar rule ended in 1921 with a coup d'etat that brought Reza Khan to power who continued with efforts to settle the nomadic tribes until WW II. His son Mohammad Reza Shah was disposed in the 1979 Iranian Revolution.

Iranian ethnic groups included in this study.

The eleven groups comprised in this study identify themselves as members of distinct ethnic groups, referring to shared ancestry, language and territory (see Fig. S1 for an ethnic overview

map of Iran). As the short overview has already demonstrated, these identifications remain social constructs that gloss over the historical complexities and voids of knowledge within the groups' historiography ([7], p. 41-44). *Iranian Arabs* speak a Semitic language and predominantly live in Khuzestan (southwestern Iran) and along the Persian Gulf coast, but are also scattered in central and eastern Iran. The settlement of Arab tribes in Iran is variously attested since the Sassanian period [8]. They became an admixed population by intermingling with other ethnic groups from the same regions, such as Persians, Turks and Lurs [9]. *Iranian Azeris* (also: Azari or Azerbaijani) mostly reside in the Northwest of Iran in an area spanned by the Caspian Sea in the east, and Lake Urmia in the west, the border to the Republic of Azerbaijan in the north and up to the latitude of Tehran southwards. This area has been a passageway of human migrations for thousands of years. Azeris speak a Turkic language and are often grouped together with Uzbeks and Turkmen, however, their origin remains disputed. Descent from Central Asian invaders, Indo-European ancestry with Turkic language imposition [10, 11] as well as admixture from several source populations have all been proposed. Azeris are usually grouped with the Western Turks or Seljuks who began the first Turkic migration into Iran, but these are not their sole ancestry as local populations adopted the Turkic language and later mobile Turkic groups mingled with them [12]. *Iranian Baluchis* (also: Balochs) speak an Iranian (Indo-European; IE) language of the Northwest Iranian language family. Historically, their migration from northwestern Iran is attested in the Timurid period (but see [13], p. 270-271, for a critical review of the concept of "being Baluchi"). They predominantly reside in the southeast of Iran (Baluchistan), stretching from the Pakistani and Afghan borders to the Gulf of Oman, again an area that has experienced human migrations in the past millennia. *Iranian Kurds* also speak a variety of languages belonging to the southwest Iranian language group. They predominantly reside in the northwest and west of Iran close to the borders to Turkey and Iraq (Kurdistan) as well as in the extreme northeastern province of Khorassan around the city of Ashkhane. As the name kurd seems to derive from a generic denominator for mobile herders, they probably derive from diverse ancestry ([5], p. 120). Iranian *Gilaks* (also: Gilani) and *Mazanderanis* (also: Mazani, Tabari) are also speakers of IE languages and have their predominant residence at the southwestern coast of the Caspian Sea around the city of Rasht and at the Southern and Southeastern Caspian Sea coast north of the Alborz mountains, respectively. *Lurs* also use an IE language and inhabit the mountainous areas of the northern Fars province and the southern Zagros in West Iran (Luristan). *Iranian Persians* (also: Farsi) as the largest ethnic group, speaking Indo-Iranian Farsi. They inhabit the central and eastern parts of Iran, including the

major provinces Tehran, Isfahan, Kerman, Yazd and Fars. Gilakis, Mazanderanis and Sistanis (see below) are considered to be very closely related to Persians and are actually often treated as one single group. *Persian Gulf (PG) Islanders* reside on islands in the Persian Gulf, such as Qeshm Island. This special situation has led to both isolation from mainland Iran, and exposure to potential admixture through contact with seafaring people, including African slaves that were traded to Iran until 1848 ([14], p. 351-2). *Iranian Sistanis* reside in the Sistan, representing the northern part of the "Sistan and Baluchistan" province and bordering Afghanistan on the North and East, and Lake Hamun on the West. They speak an Iranian language classified as western Baluchi and like those trace their origins to the northwestern region of Iran. Sistani is considered a dialect of Persian [15]. Finally, *Iranian Turkmen* (or Turkoman) speak a Turkic language classified as East Turkic and have their predominant residence at the eastern coast of the Caspian Sea in the extreme north of Iran bordering Turkmenistan. They are thought to be of Central Asian ancestry.

References

1. Witzel M. Iranian migration. In: Potts DT, editor. The Oxford Handbook of Ancient Iran. Oxford: Oxford University Press; 2013. p. 423-41.
2. Anthony DW. The Horse, the wheel, and language. How Bronze Age riders from the Eurasian steppes shaped the Modern World. Princeton, Oxford: Princeton University Press; 2007.
3. Lamberg-Karlovsky CC. Archaeology and Language: The Indo-Iranians. Current Anthropology. 2002;43(1):63-88. doi: 10.1086/324130.
4. Stolper MW. No. 190. In: Harper PO, Aruz J, editors. The royal city of Susa: Ancient Near Eastern treasures in the Louvre. New York: The Metropolitan Museum of Art; 1992. p. 271-72.
5. Potts DT. Nomadism in Iran: from antiquity to the modern era. New York: Oxford University Press; 2014.
6. Okazaki S. The Great Persian Famine of 1870-71. Bulletin of the School of Oriental and African Studies, University of London. 1986;49(1):183-92.
7. Beck L. The Qashqa'i of Iran. New Haven: Yale University Press; 1986.
8. Oberling P, Hourcade B. ʿARAB iv. Arab tribes of Iran – Encyclopaedia Iranica. In: Yarshater E, editor. Encyclopedia Iranica. 21986. p. 215-20.
9. Rashidvash V. Iranian people: Iranian ethnic groups. Int J Humanities and Soc Sciences. 2013;3(15):216-26.
10. Rashidvash V. Iranian people and the race of people settled in the Iranian plateau. Int J Humanities and Soc Science Studies. 2016;III(I):181-91.
11. Yunusbayev B, Metspalu M, Metspalu E, Valeev A, Litvinov S, Valiev R, et al. The genetic legacy of the expansion of Turkic-speaking nomads across Eurasia. PLoS Genet. 2015;11(4):e1005068. doi: 10.1371/journal.pgen.1005068. PubMed PMID: 25898006; PubMed Central PMCID: PMCPMC4405460.
12. Oberling P. The Tribes of Qarāca Dāġ: A Brief History. Oriens. 1964;17:60-95. doi: 10.2307/1580019.
13. Jahani C. The Baloch as an Ethnic Group in the Persian Gulf Region. The Persian Gulf in Modern Times. New York: Palgrave Macmillan; 2014. p. 267–97.
14. Mirzai BA. The 1848 Abolitionist Farman: A Step towards Ending the Slave Trade in Iran. In: Campbell G, editor. Abolition and Its Aftermath in the Indian Ocean Africa and Asia. London: Routledge; 2005. p. 94–102.
15. Veryho JW. Sistani-Persian Folklore. Indo-Iranian Journal 1962;5:276-307. doi: 10.1163/000000062791616110
