# Supplementary figures and images for: Distinct genetic variation and heterogeneity of the Iranian population
Source: PLoS Genet. 2019 Sep 24;15(9):e1008385. doi: 10.1371/journal.pgen.1008385 (PMC6759149; doi:10.1371/journal.pgen.1008385)

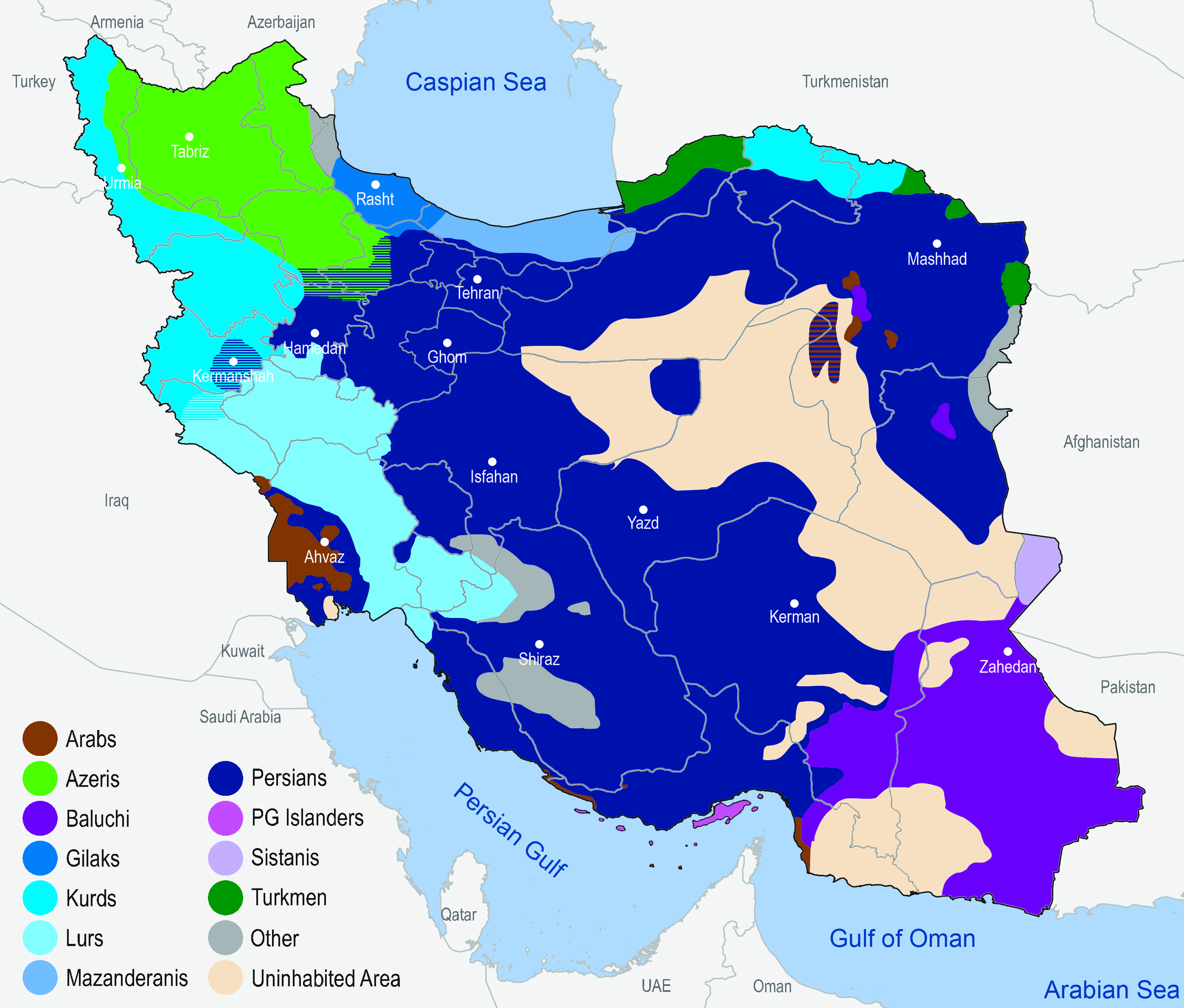

Supplement: S1 Fig — Regions with predominance of a particular ethnic group are designated by color. Adapted, modified and simplified from [8] and http://legacy.lib.utexas.edu/maps/iran.html (Perry-Castañeda Library Map Collection, The University of Texas at Austin, USA; file iran_country_profile_2009.jpg). (TIF) [file pgen.1008385.s002.tif]

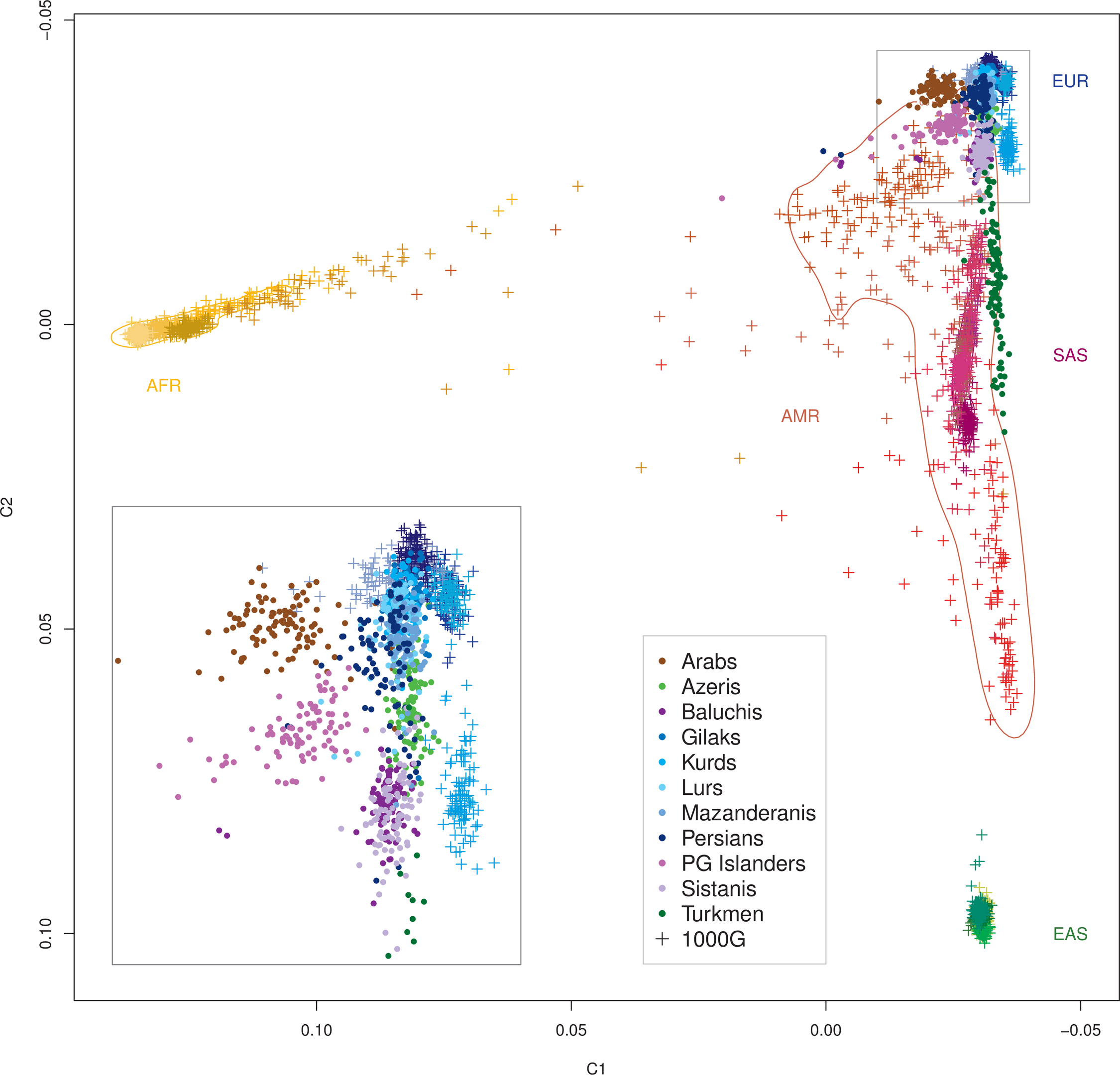

Supplement: S2 Fig — Relative sample locations with respect to the first two MDS components. Iranian ethnic groups in a global context (global 1000G data set, with 90% density limits); inlet: zoomed view of the CIC and adjacent European populations. (TIF) [file pgen.1008385.s003.tif]

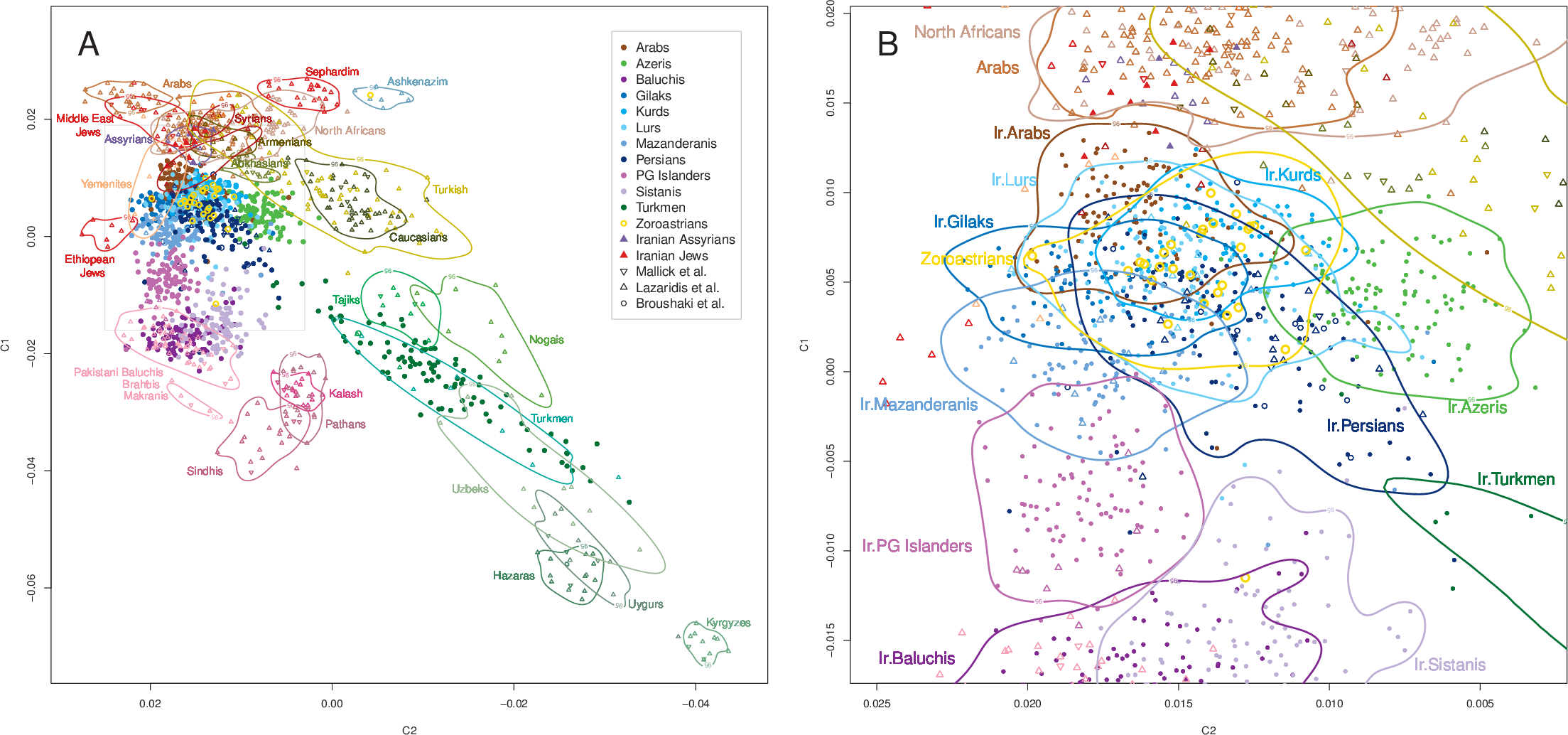

Supplement: S3 Fig — A. Regional context. Relative sample locations in the first two MDS components based on the samples from this study and the local reference data set, including 90% density limits. European samples were omitted for clarity. B. Zoomed view of (A) to the CIC and adjacent groups. (TIF) [file pgen.1008385.s004.tif]

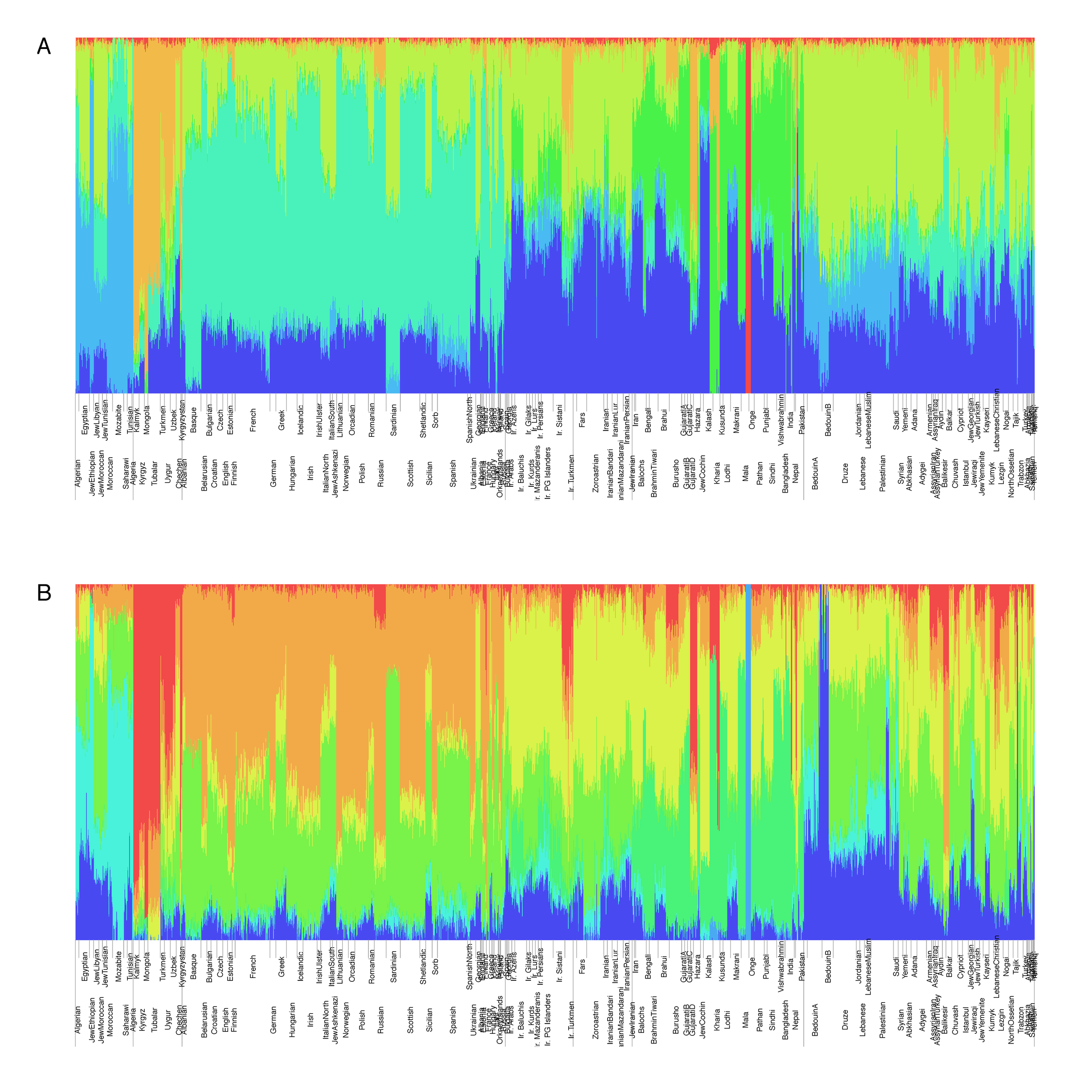

Supplement: S4 Fig — Inference was based on the local data set, but with the Iranian samples randomly down-sampled to assess a potentially biasing effect of the comparatively larger sample sizes compared to the other populations from the region. For each of the seven CIC ethnic group, 5 samples were randomly drawn, whereas the other four non-CIC groups contributed 20 random samples each. Given are the solutions providing the smallest errors. A: k = 7, CV = 0.57390; B: k = 8, CV = 0.57393. (TIF) [file pgen.1008385.s005.tif]

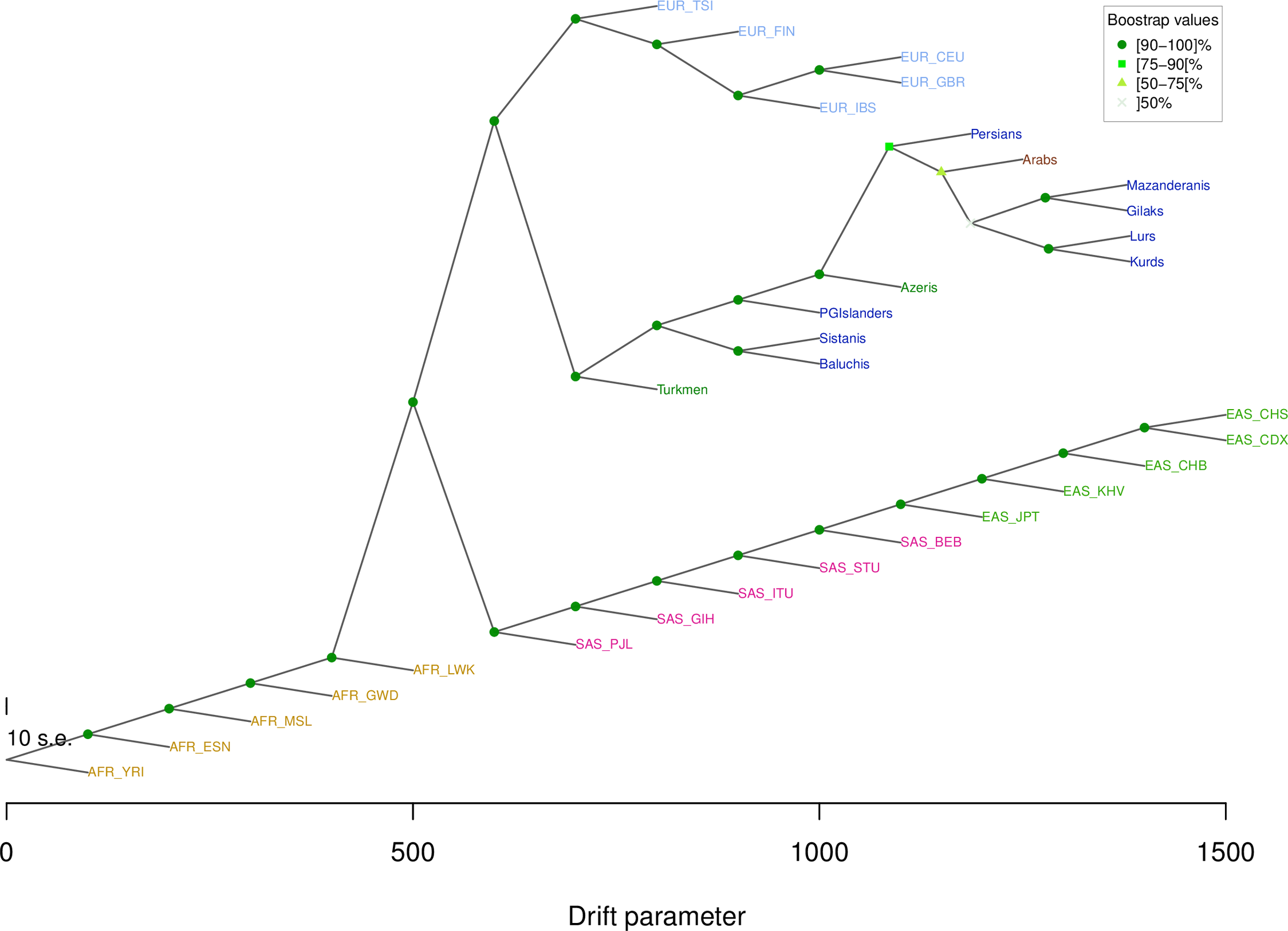

Supplement: S5 Fig — Nodes are colored by bootstrapping support, edges by weight of migration. (TIF) [file pgen.1008385.s006.tif]

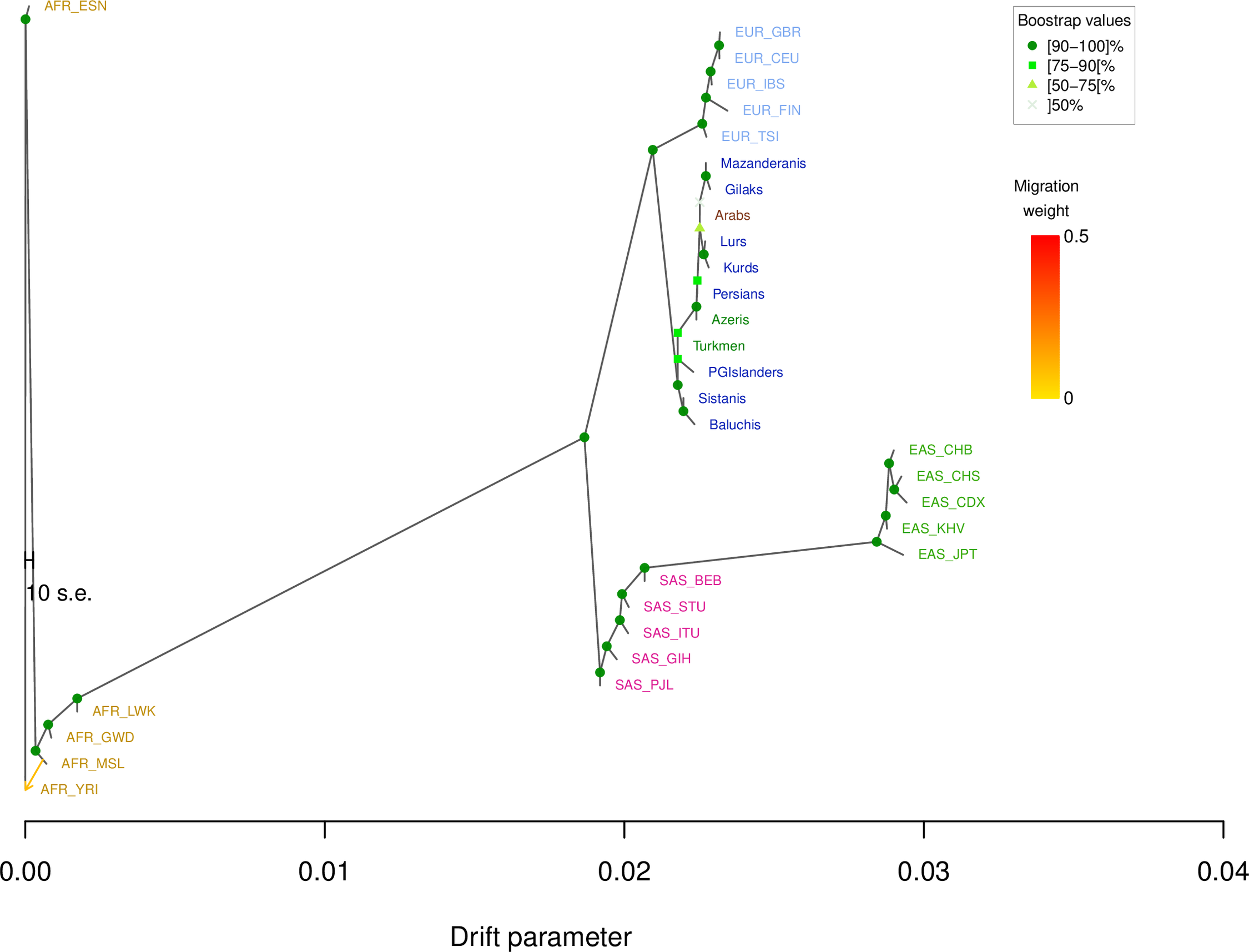

Supplement: S6 Fig — Nodes are colored by bootstrapping support, edges by weight of migration. (TIF) [file pgen.1008385.s007.tif]

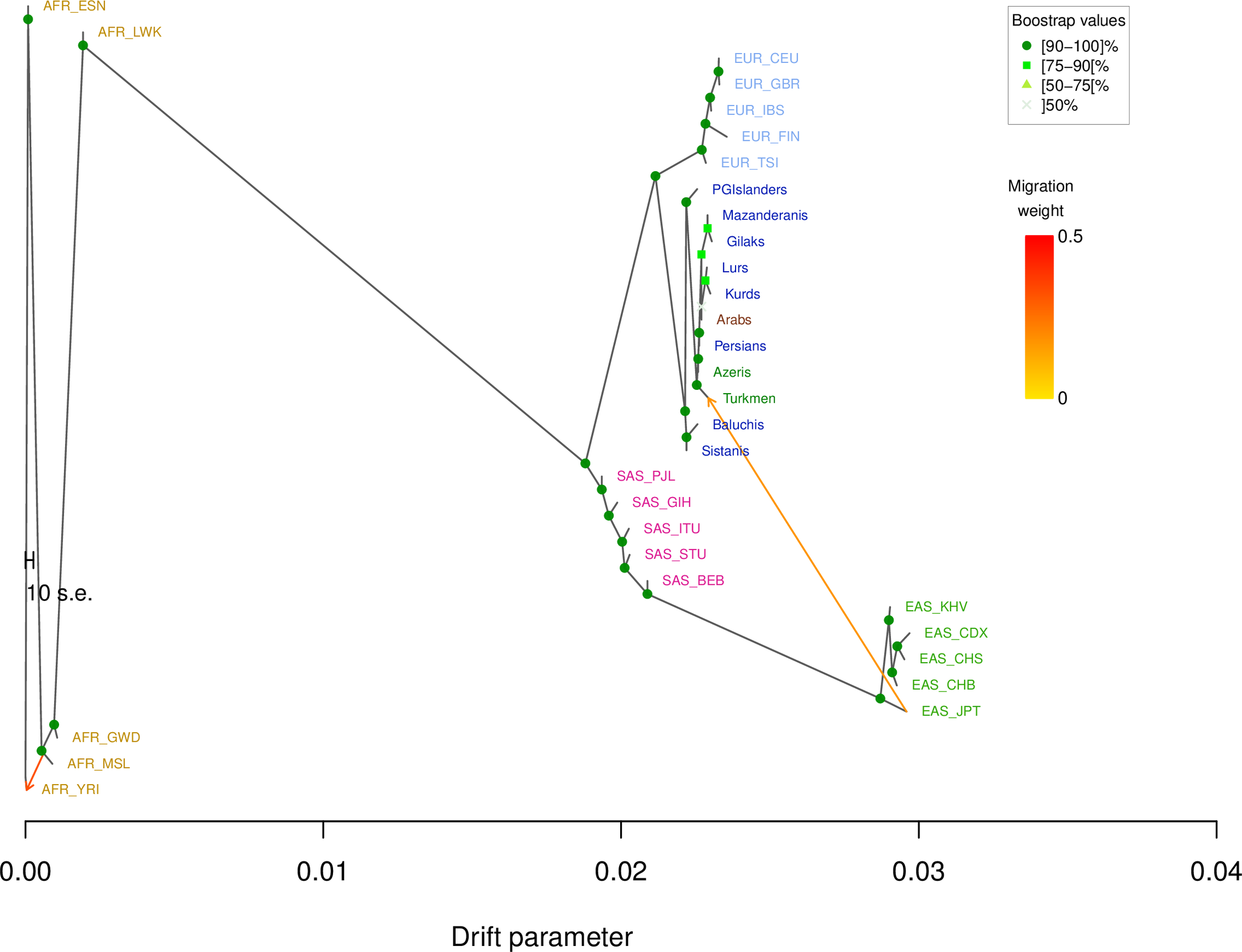

Supplement: S7 Fig — Nodes are colored by bootstrapping support, edges by weight of migration. (TIF) [file pgen.1008385.s008.tif]

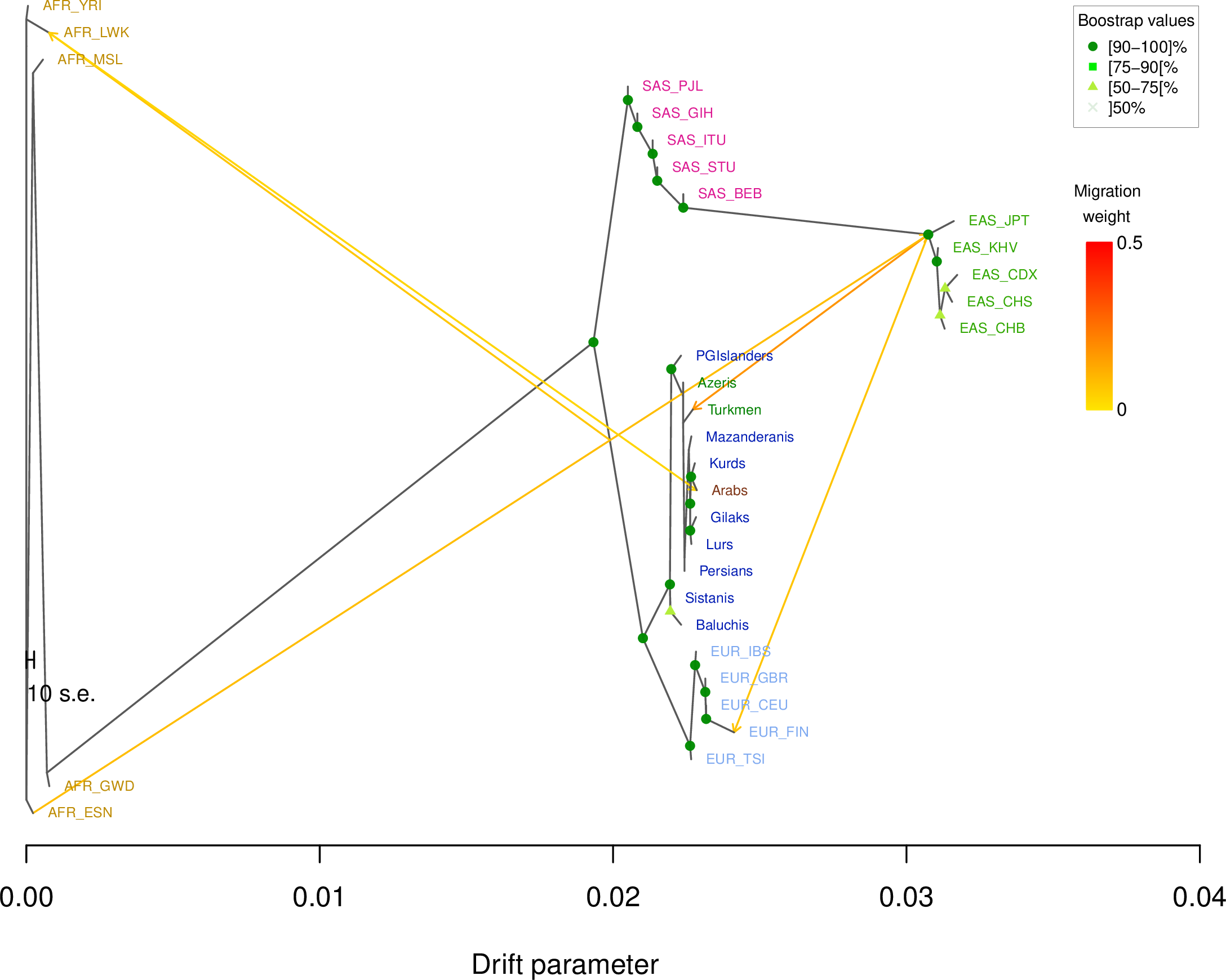

Supplement: S8 Fig — Nodes are colored by bootstrapping support, edges by weight of migration. (TIF) [file pgen.1008385.s009.tif]

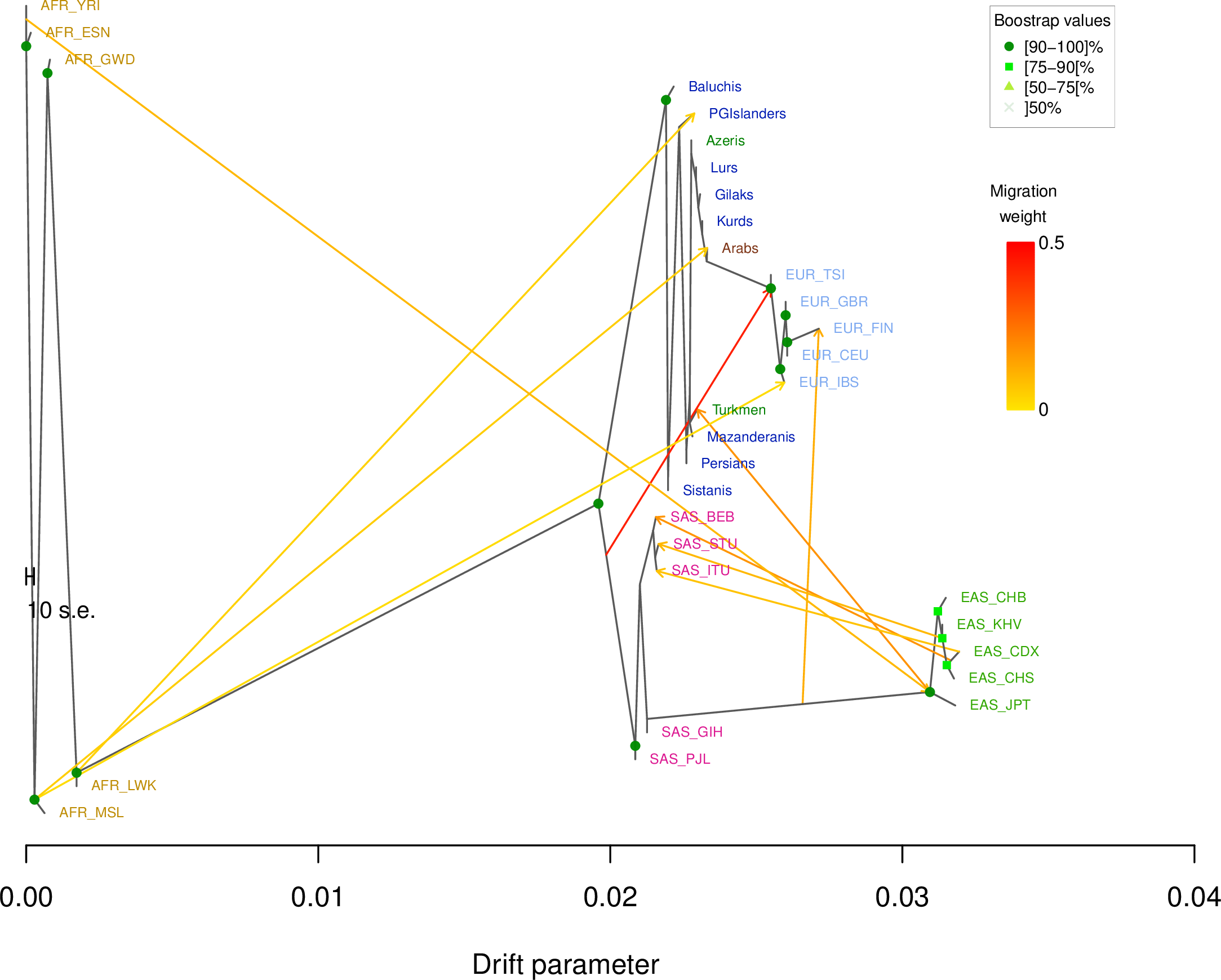

Supplement: S9 Fig — Nodes are colored by bootstrapping support, edges by weight of migration. (TIF) [file pgen.1008385.s010.tif]

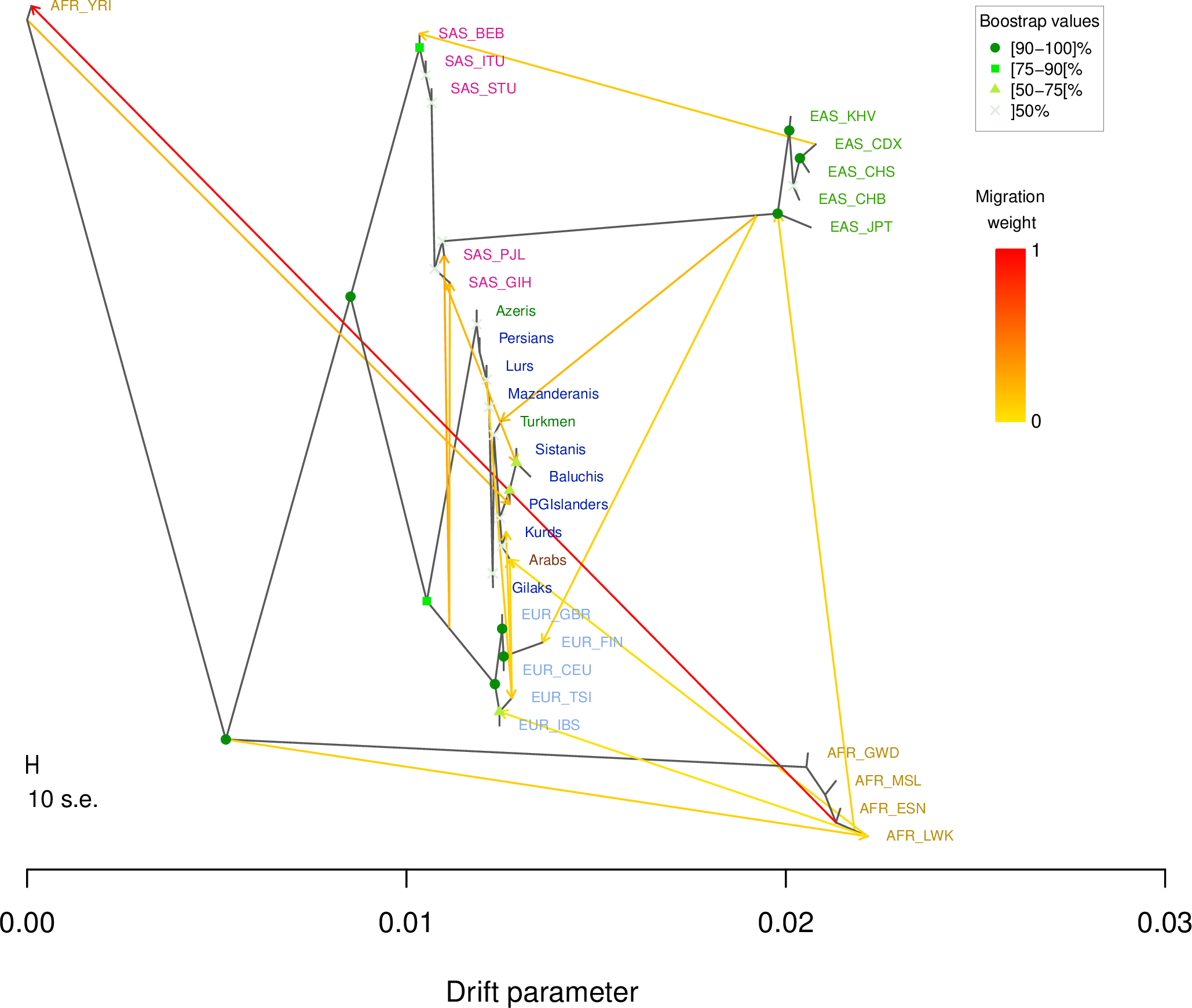

Supplement: S10 Fig — Nodes are colored by bootstrapping support, edges by weight of migration. (TIF) [file pgen.1008385.s011.tif]

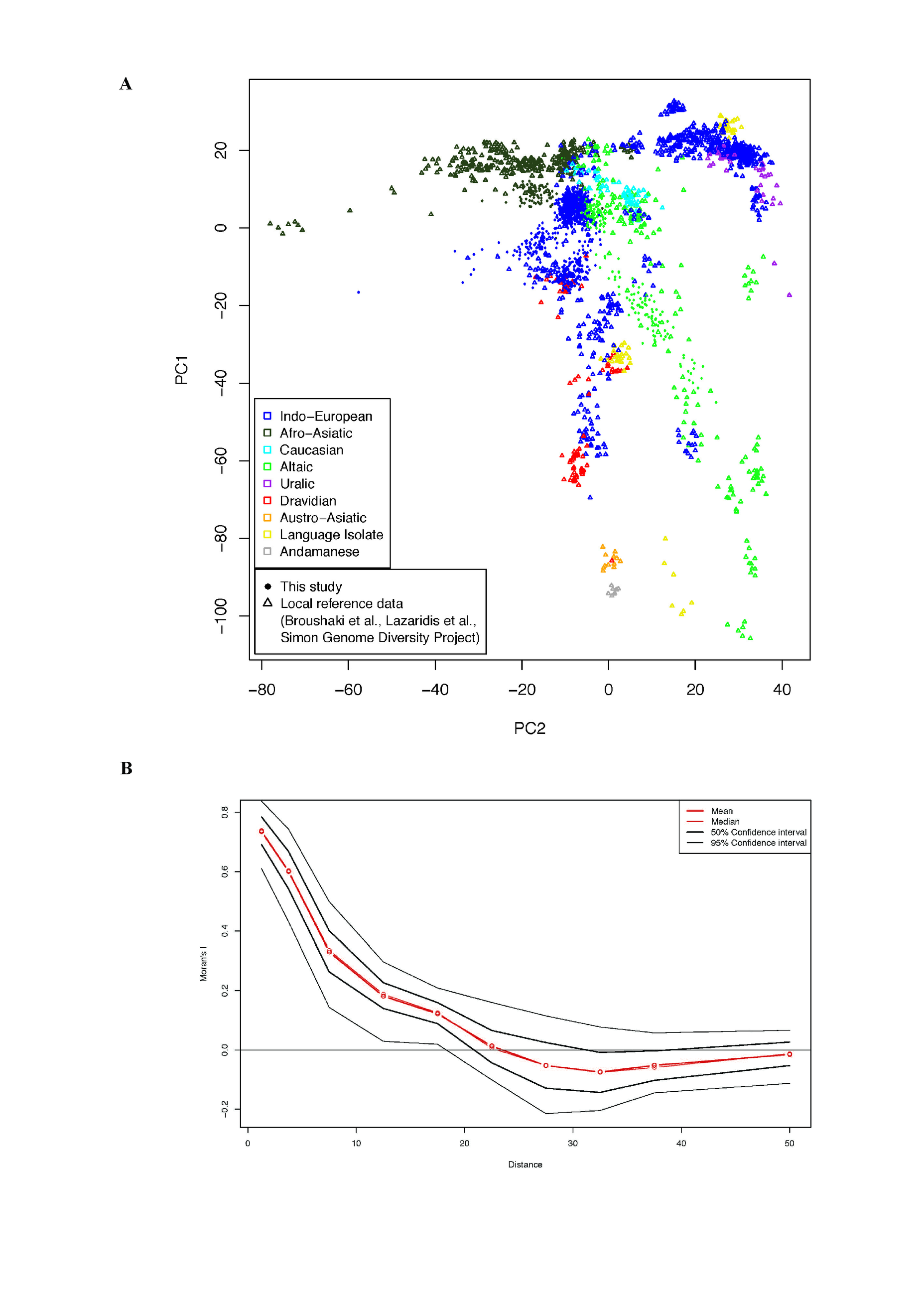

Supplement: S11 Fig — A. PCA plot of the local data set, colored by spoken language family. See S2 Table for language assignments. B. Autocorrelation analysis. Distance between pairs of individuals defined by the Euclidean distance with respect to the first two principal components. (TIF) [file pgen.1008385.s012.tif]

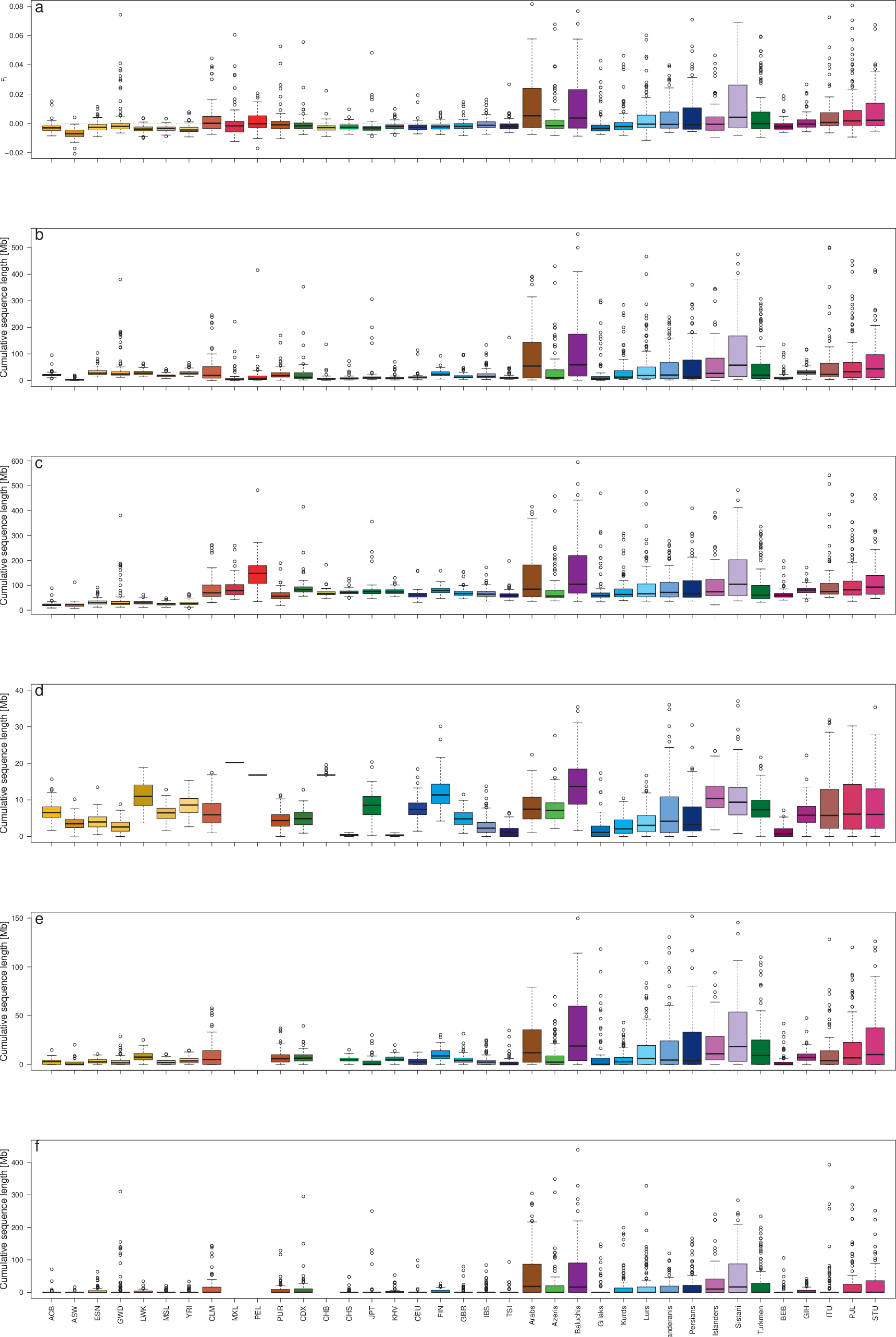

Supplement: S12 Fig — A. Inbreeding coefficient FI; B. IBDseq-defined autozygous regions (HBD). Total genomic autozygous sequence [Mb]; C. PLINK-defined runs of homozygosity (ROHs). Total genomic sequence [Mb] of ROHs; D-F. GARLIC-defined ROHs. Total genomic sequence [Mb] located in class A short and likely ancient ROHs (D), class B intermediate-length ROHs (E) and class C long and likely recent ROHs (F), respectively. (TIF) [file pgen.1008385.s013.tif]

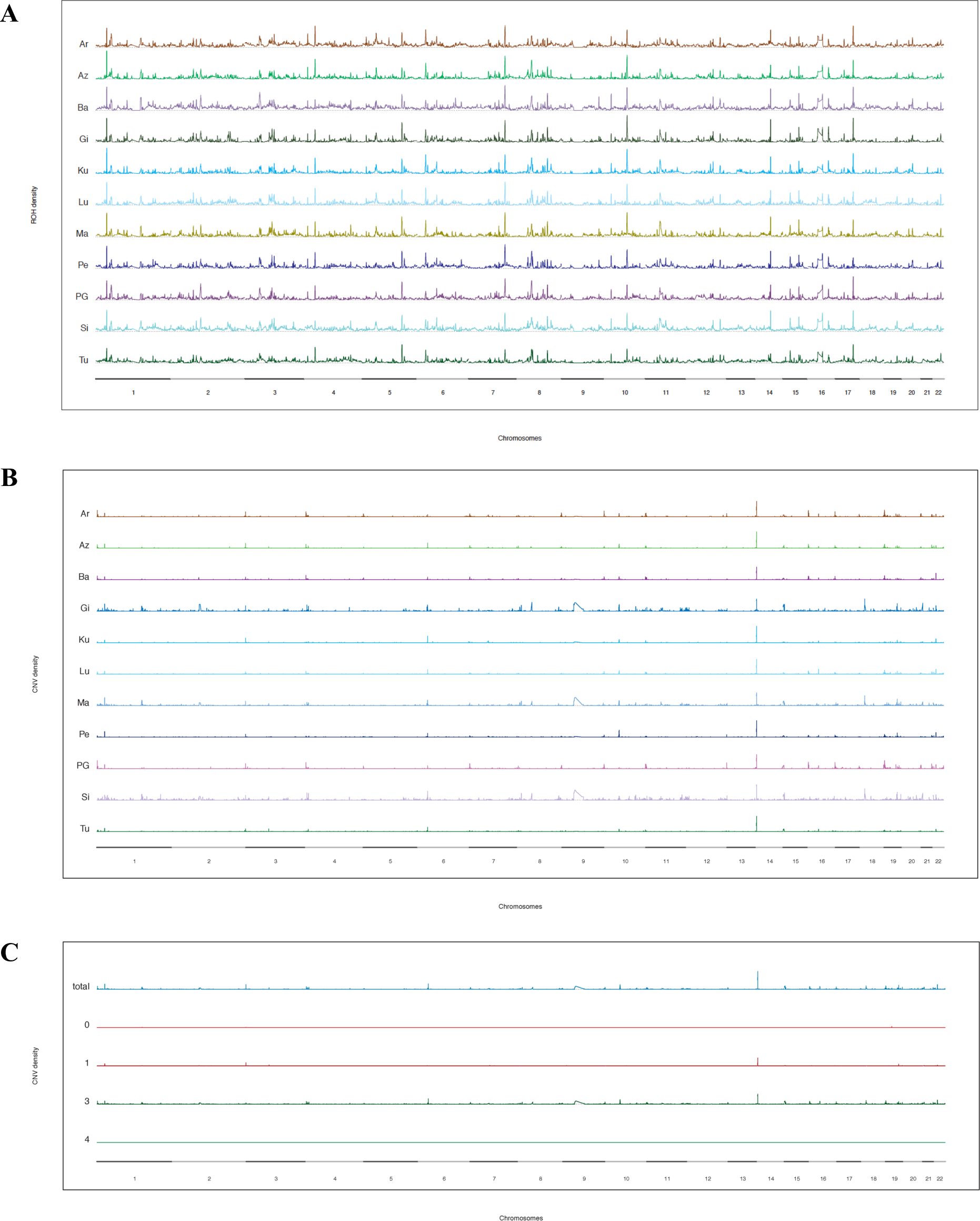

Supplement: S13 Fig — A. Density of PLINK-defined runs of homozygosity (ROHs). Sample proportion per group featuring an ROH at a given genomic location. B. Density of copy-number variation (CNV). Sample proportion per group featuring any of four CNV types at a given genomic location. C. CNV type density in the Iranian population. Total: cumulative portion over all four CNV types; 0: loss of both copies; 1: loss of one copy; 3: gain of one copy; 4: gain of two copies. (TIF) [file pgen.1008385.s014.tif]

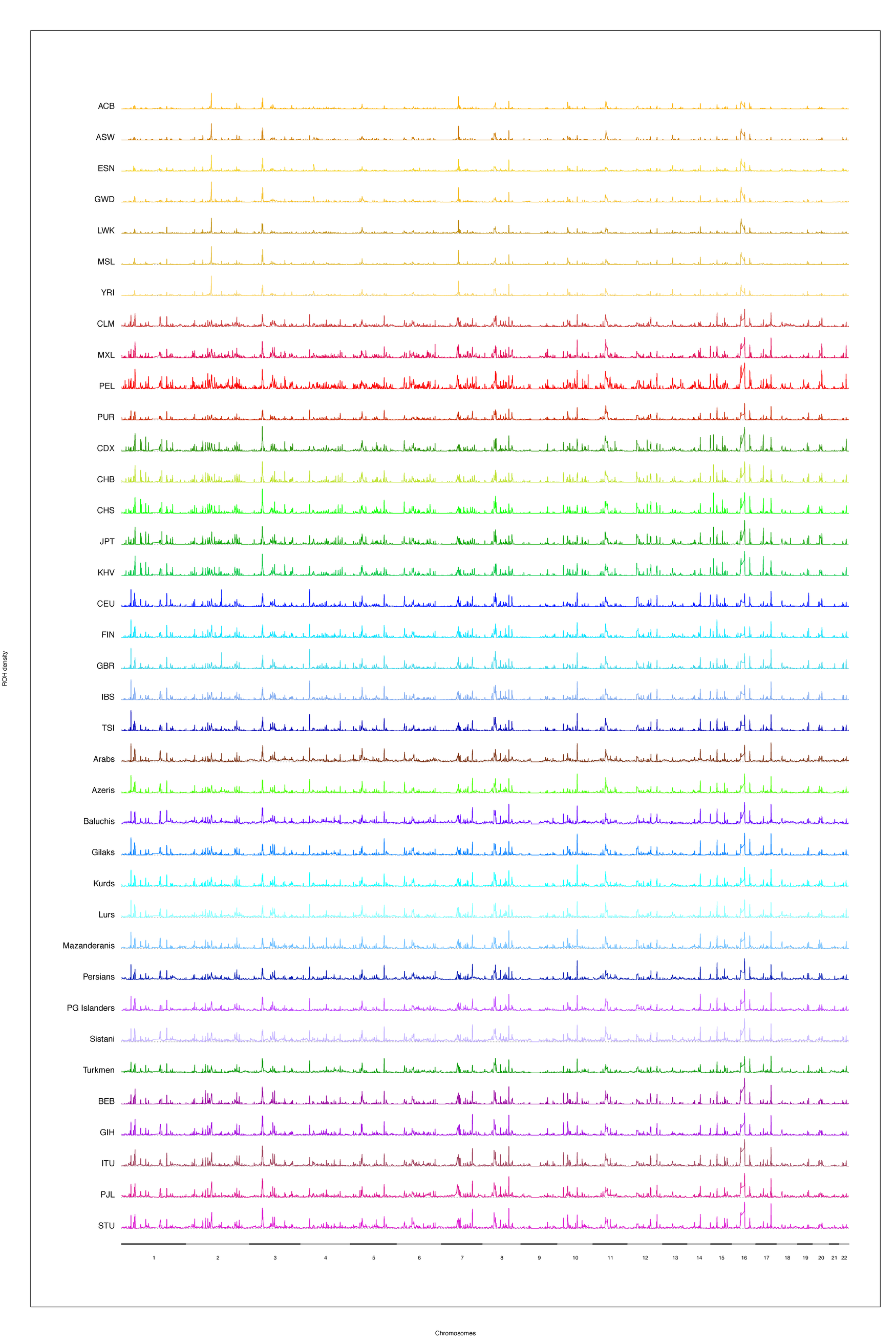

Supplement: S14 Fig — Sample proportion per group featuring an ROH at a given genomic location. (TIF) [file pgen.1008385.s015.tif]

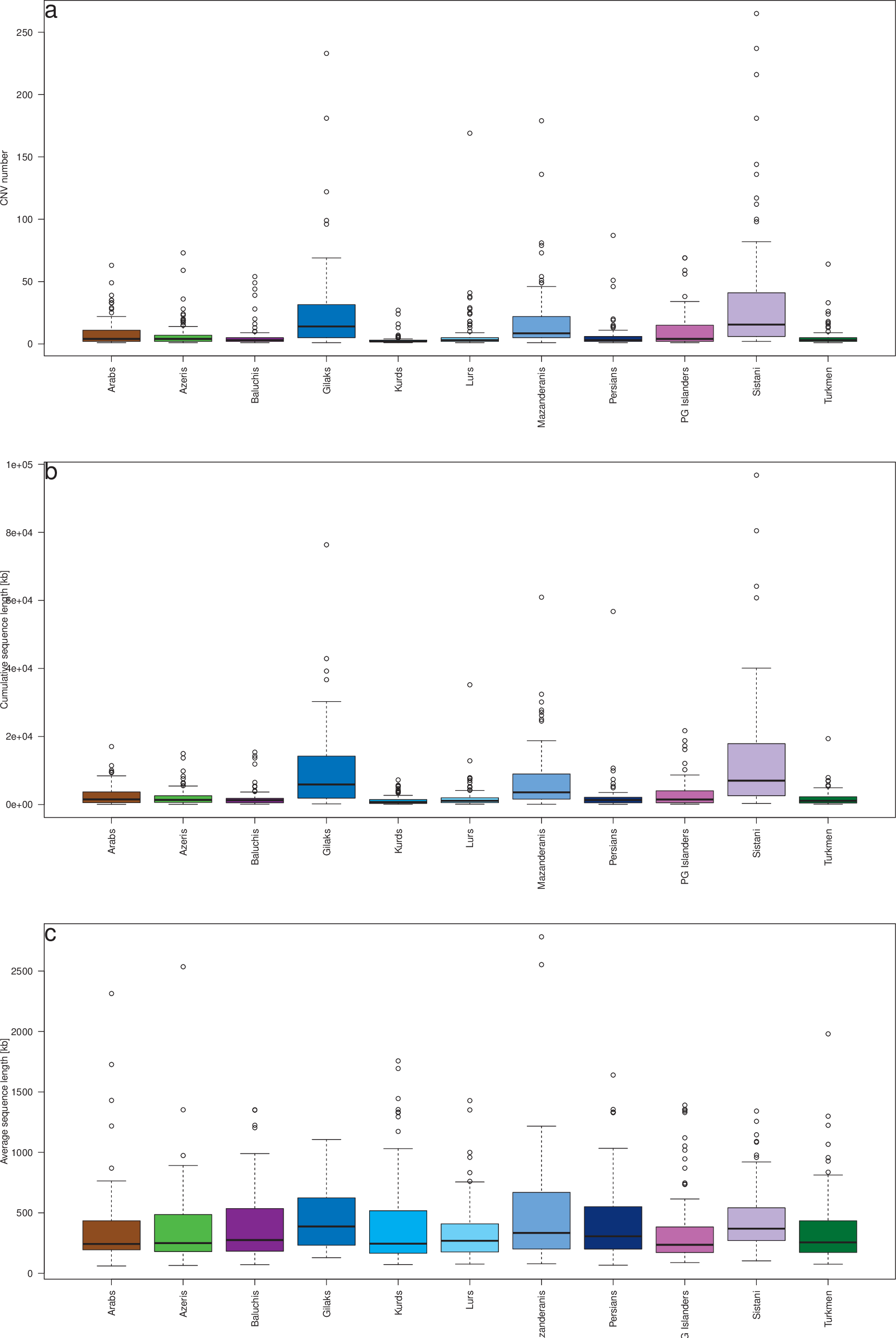

Supplement: S15 Fig — A. Number of detected CNVs per group. B. Cumulative sequence length [kb], i.e. total length of genomic sequence included in CNVs. C. Average sequence length per CNV [kb]. (TIF) [file pgen.1008385.s016.tif]

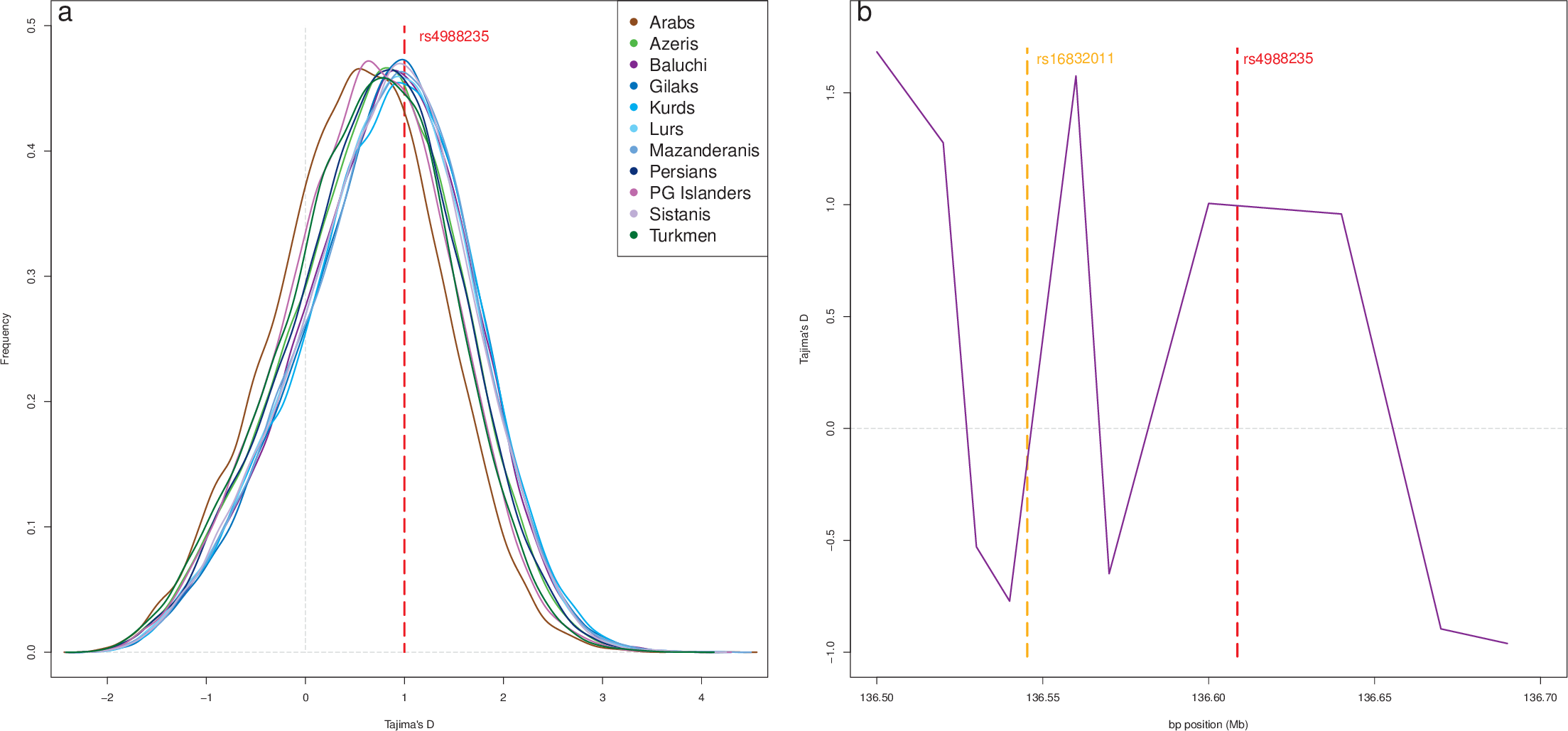

Supplement: S16 Fig — A. Autosome-wide distribution density of Tajima’s D (100 kb window size), separately for each Iranian ethnic group. B. Local Tajima’s D values around rs4988235 (100 kb window size; 10 kb shift) in Baluchis. Red line: rs4988235; orange line: rs16832011. (TIF) [file pgen.1008385.s017.tif]

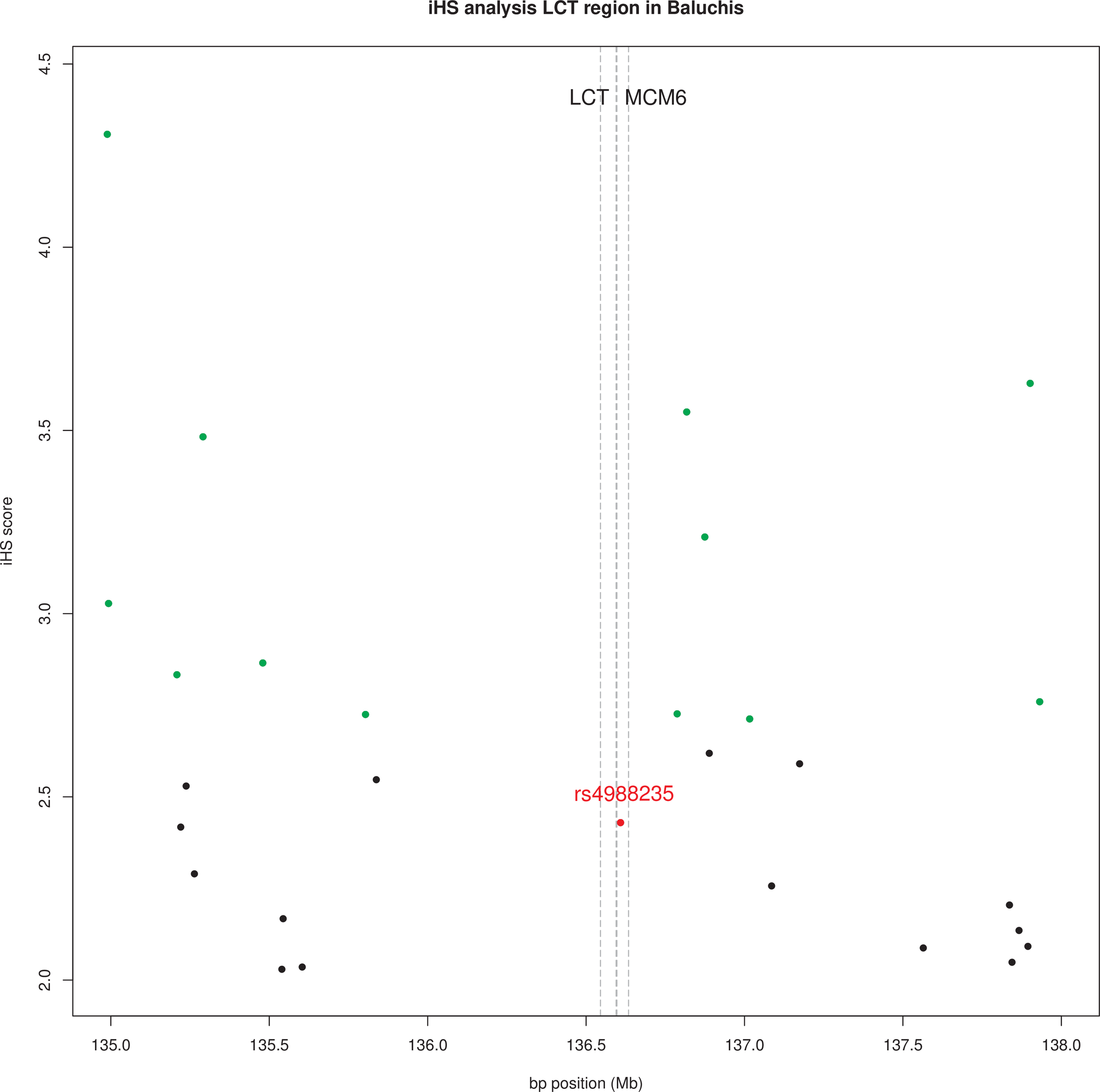

Supplement: S17 Fig — Shown are extreme absolute iHS values on chromosome 2 around the LCT gene region. Black dots: SNPs with |iHS|>2; green dots: SNPs with |iHS|>2.63, there representing the top 1% of SNPs on chromosome 2 with the largest absolute iHS values (in line with [115]); red dot: lactase persistence SNP rs4988235 (iHS = -2.51); grey dashed lines: borders of the LCT (136.54–136.59 Mb) and MCM6 (136.59–136.63 Mb) gene regions according to GRCh37/hg19 (www.genecards.org; [116]). (TIF) [file pgen.1008385.s018.tif]

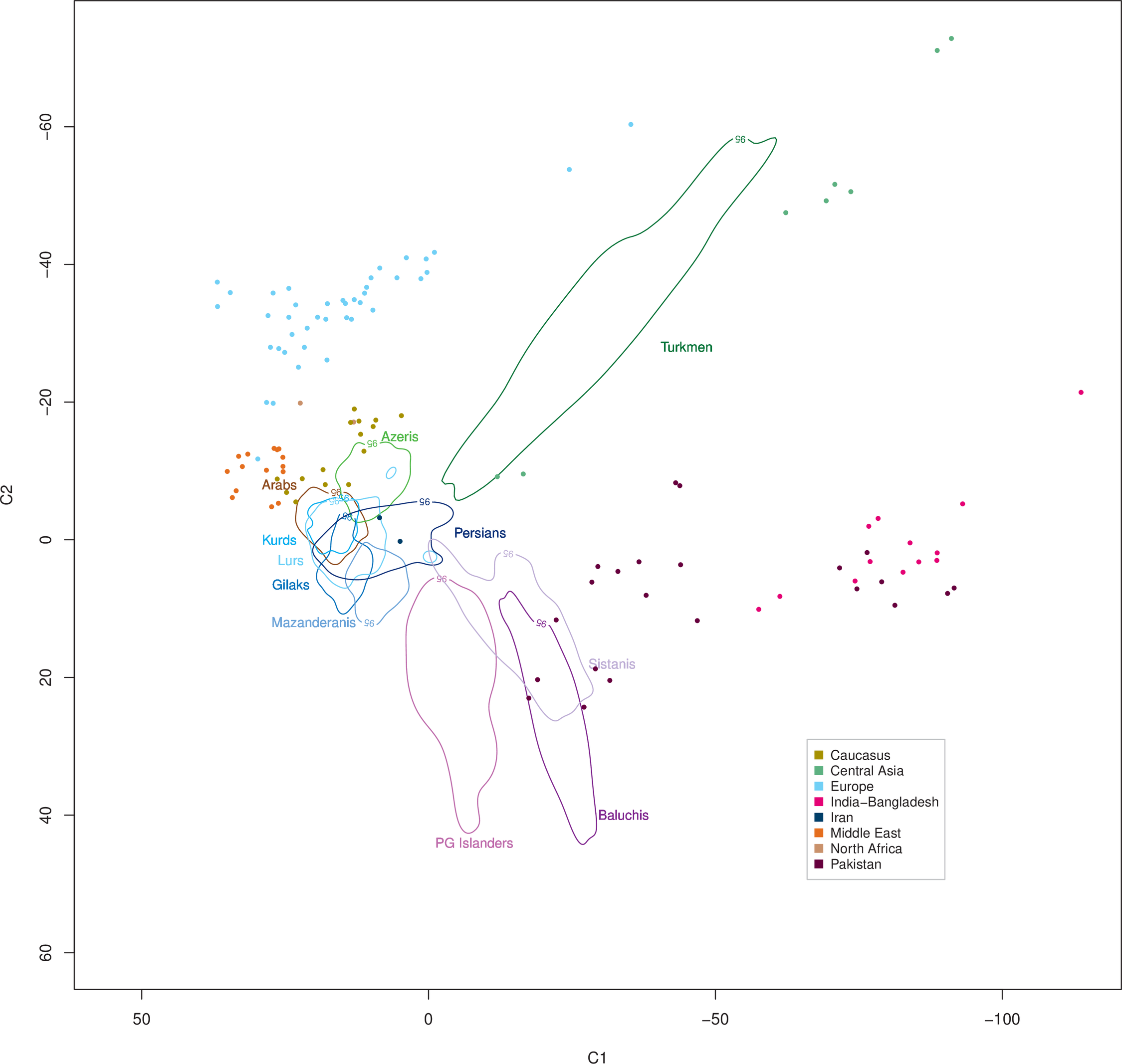

Supplement: S18 Fig — First two MDS components defined by the Iranian groups from this study (indicated by 95% density limits) and selected SGDP [44] samples from the region (S4 Table; dots). (TIF) [file pgen.1008385.s019.tif]
