# Supplementary material for: Distinct genetic variation and heterogeneity of the Iranian population
Source: PLoS Genet. 2019 Sep 24;15(9):e1008385. doi: 10.1371/journal.pgen.1008385 (PMC6759149; doi:10.1371/journal.pgen.1008385)
Supplement: S1 Table — Samples from selected ethnic groups or sampling sites from [2, 44] were assembled to supergroups for better visualization. (DOCX) [file pgen.1008385.s020.docx]

**S1 Table. Assignment of reference samples to population supergroups in the local data set.** Samples from selected ethnic groups or sampling sites were assembled to supergroups for better visualization.

| **Supergroup** | **Abbreviation** | **Samples** |
| --- | --- | --- |
| Caucasians / Turkish | CAU | Abkhasian, Adana, Adygei, Armenian, Aydin, Balikesir, Balkar, Chechen, Georgian, Georgian Jew, Kayseri, Kumyk, Lezgin, North Ossetian, Trabzon, Turkey, Turkey Assyrian |
| Central Asians | CAS | Hazara, Kalmyk, Kyrgyz, Mongola, Nogai, Tajik, Tubalar, Turkmen, Uygur, Uzbek |
| Europeans | EUR | Albanian, Ashkenazi Jew, Basque, Belarusian, Bulgarian, Crete, Croatian, Czech, English, Estonian, Finnish, French, German, Greek, Hungarian, Icelandic, Irish, Irish Ulster, Italian North, Italian South, Lithuanian, Norwegian, Orcadian, Polish, Romanian, Russian, Saami, Sardinian, Scottish, Shetlandic, Sicilian, Sorb, Spanish, Spanish North, Ukrainian |
| Iranians | IRI | Iranian, Iranian Assyrian, Iranian-Bandar Abbas, Iranian Jew, Lur, Mazanderani, Persian |
| Middle Easterners | MDE | BedouinA, BedouinB, Cypriot, Druze, Jordanian, Iraqi Assyrian, Iraqi Jew, Lebanese, Lebanese Christian, Palestinian, Samaritan, Saudi, Syrian, Yemeni, Yemenite Jew |
| North Africans | AFR | Algerian, Egyptian, Moroccan, Mozabite, Saharawi |
| Sephardim | SPH | Libyan Jew, Moroccan Jew, Tunisian Jew, Turkey Jew |
| South Asians | SAS | Bengali, Brahmin Tiwari, Brahui, Burusho, GujaratiA-D, Chochin Jew, Irula, Kalash, Kapu, Kharia, Khondadora, Kusunda, Lodhi, Madiga, Makrani, Mala, Onge, Pakistani Baluchi, Pathan, Punjabi, Reli, Sindhi, Yadava, Vishwabrahmin |
