# Supplementary material for: Distinct genetic variation and heterogeneity of the Iranian population
Source: PLoS Genet. 2019 Sep 24;15(9):e1008385. doi: 10.1371/journal.pgen.1008385 (PMC6759149; doi:10.1371/journal.pgen.1008385)
Supplement: S2 Table — *: Present in this study’s data set. Assignment has been based in the following sources: https://www.ethnologue.com/browse/countries; http://glottolog.org/; http://wals.info/languoid. (DOCX) [file pgen.1008385.s021.docx]

**S2 Table. Language family assignment of ethnic groups in the local data set.** *: Present in this study’s data set. Assignment has been based in the following sources: https://www.ethnologue.com/browse/countries; http://glottolog.org/; http://wals.info/languoid

| **Language family** | **Ethnic groups** |
| --- | --- |
| Indo-European | Albanian, Armenian, Baluch*, Belarusian, Bengali, Bulgarian, Crete, Croatian, Cypriot, Czech, English, French, German, Greek, Gujarati, Hazara, Icelandic, Iranian (Bandar Abbas, Farsi, Gilak*, Iranian Jew, Kurd*, Lur*, Mazandarani*, Persian*, PG Islander*, Sistani*, Zoroastrian), Irish (Irish Ulster), Italian, Kalash, Lithuanian, Lodhi, Makrani, Norwegian, Orcadian, Ossetian, Pathan, Polish, Punjabi, Relli, Romanian, Russian, Sardinian, Scottish (Shetlandic), Sicilian, Sindhi, Sorb, Spanish, Tajik, Ukrainian |
| Afro-Asiatic | Algerian, Assyrian (Iraqi, Iranian, Turkish), Bedouin, Druze, Egyptian, Iranian Arab*, Ashkenazi Jew, Ethiopian Jew, Iraqi Jew, Libyan Jew, Moroccan Jew, Tunisian Jew, Yemenite Jew, Jordanian, Lebanese (Christian, Muslim), Moroccan, Mozabite, Palestinian, Saharawi, Samaritan, Saudi, Syrian, Tunisian, Yemeni |
| Uralic | Estonian, Finnish, Hungarian, Saami |
| Altaic | Azeris*, Balkar, Chuvash, Kalmyk, Kumyk, Kyrgyz, Mongola, Tubalar, Turkish (Adana, Aydin, Balikesir, Istanbul, Turkish Jew, Kayseri, Nogai, Trabzon), Turkmen*, Uygur, Uzbek |
| Caucasian | Abkhasian, Adygei, Chechen, Georgian, Georgian Jew, Lezgin |
| Dravidian | Brahmin (BrahminTiwari, Vishwabrahmin), Brahui, Irula, Cochin Jew, Kapu, KhondaDora, Madiga, Mala, Yadava |
| Austro-Asiatic | Kharia |
| Andamanese | Onge |
| Language Isolates | Basque, Burusho, Kusunda |
