# Supplementary material for: Distinct genetic variation and heterogeneity of the Iranian population
Source: PLoS Genet. 2019 Sep 24;15(9):e1008385. doi: 10.1371/journal.pgen.1008385 (PMC6759149; doi:10.1371/journal.pgen.1008385)
Supplement: S4 Table — (DOCX) [file pgen.1008385.s023.docx]

**S4 Table. Assignment of SGDP reference samples to population supergroups in the aDNA plot.**

| **Group** | **Samples** |
| --- | --- |
| Caucasus | Abkhasian, Adygei, Armenian, Chechen, Georgian, Lezgin, North Ossetian, Turkey |
| Central Asia | Hazara, Kyrgyz, Tajik, Uygur |
| Europe | Albanian, Bulgarian, Crete, Czech, English, Estonian, Finnish, French, Greek, Hungarian, Icelandic, Italian, Norwegian, Orcadian, Polish, Russian, Saami, Spanish, Spanish North, Ukrainian |
| India-Bangladesh | Bengali, Brahmin, Irula, Kapu, Khondadora, Madiga, Reli |
| Iran | Iranian |
| Middle East | BedouinB, Druze, Iraqi Jew, Jordanian, Palestinian, Samaritan, Yemenite Jew |
| North Africa | Algerian |
| Pakistan | Brahui, Burusho, Kalash, Makrani, Mala, Pakistani Baluchi, Pathan, Punjabi, Sindhi, Yadava |
