# Supplementary material for: Distinct genetic variation and heterogeneity of the Iranian population
Source: PLoS Genet. 2019 Sep 24;15(9):e1008385. doi: 10.1371/journal.pgen.1008385 (PMC6759149; doi:10.1371/journal.pgen.1008385)
Supplement: S5 Table — (DOCX) [file pgen.1008385.s024.docx]

**S5 Table. Weir’s F_ST_ for pairs of an Iranian ethnic group and a 1000 Genomes population.**

| **Continent** | **Population** | **Iranian Arabs** | **Iranian Azeris** | **Iranian Baluchis** | **Iranian Gilaks** | **Iranian Kurds** | **Iranian Lurs** | **Iranian Mazanderanis** | **Iranian Persians** | **Iranian PG Islanders** | **Iranian Sistanis** | **Iranian Turkmen** |
| --- | --- | --- | --- | --- | --- | --- | --- | --- | --- | --- | --- | --- |
| **AFR** | **ACB** | 0.0999 | 0.1084 | 0.1081 | 0.1119 | 0.1107 | 0.1094 | 0.1105 | 0.1064 | 0.1011 | 0.1066 | 0.1059 |
|  | **ASW** | 0.0764 | 0.0838 | 0.0841 | 0.0873 | 0.0862 | 0.0850 | 0.0860 | 0.0821 | 0.0778 | 0.0823 | 0.0809 |
|  | **ESN** | 0.1290 | 0.1386 | 0.1374 | 0.1424 | 0.1409 | 0.1395 | 0.1406 | 0.1362 | 0.1298 | 0.1362 | 0.1354 |
|  | **GWD** | 0.1242 | 0.1336 | 0.1326 | 0.1373 | 0.1358 | 0.1344 | 0.1356 | 0.1313 | 0.1252 | 0.1314 | 0.1306 |
|  | **LWK** | 0.1166 | 0.1262 | 0.1252 | 0.1298 | 0.1284 | 0.1270 | 0.1282 | 0.1239 | 0.1176 | 0.1239 | 0.1232 |
|  | **MSL** | 0.1282 | 0.1377 | 0.1365 | 0.1416 | 0.1400 | 0.1386 | 0.1398 | 0.1354 | 0.1290 | 0.1353 | 0.1345 |
|  | **YRI** | 0.1282 | 0.1378 | 0.1366 | 0.1415 | 0.1400 | 0.1386 | 0.1397 | 0.1354 | 0.1290 | 0.1354 | 0.1346 |
| **AMR** | **CLM** | 0.0214 | 0.0195 | 0.0261 | 0.0242 | 0.0228 | 0.0225 | 0.0239 | 0.0212 | 0.0253 | 0.0225 | 0.0170 |
|  | **MXL** | 0.0408 | 0.0373 | 0.0415 | 0.0431 | 0.0417 | 0.0410 | 0.0419 | 0.0392 | 0.0424 | 0.0380 | 0.0279 |
|  | **PEL** | 0.0884 | 0.0835 | 0.0849 | 0.0908 | 0.0891 | 0.0880 | 0.0884 | 0.0856 | 0.0873 | 0.0816 | 0.0667 |
|  | **PUR** | 0.0156 | 0.0153 | 0.0228 | 0.0194 | 0.0178 | 0.0177 | 0.0194 | 0.0165 | 0.0207 | 0.0193 | 0.0163 |
| **EAS** | **CDX** | 0.1015 | 0.0960 | 0.0954 | 0.1052 | 0.1035 | 0.1019 | 0.1020 | 0.0989 | 0.0986 | 0.0921 | 0.0690 |
|  | **CHB** | 0.0995 | 0.0935 | 0.0936 | 0.1032 | 0.1014 | 0.0998 | 0.1000 | 0.0968 | 0.0969 | 0.0902 | 0.0645 |
|  | **CHS** | 0.1014 | 0.0955 | 0.0953 | 0.1051 | 0.1033 | 0.1017 | 0.1018 | 0.0987 | 0.0986 | 0.0920 | 0.0669 |
|  | **JPT** | 0.1017 | 0.0957 | 0.0956 | 0.1055 | 0.1037 | 0.1020 | 0.1022 | 0.0991 | 0.0991 | 0.0923 | 0.0666 |
|  | **KHV** | 0.0974 | 0.0919 | 0.0914 | 0.1010 | 0.0994 | 0.0979 | 0.0979 | 0.0949 | 0.0946 | 0.0881 | 0.0651 |
| **EUR** | **CEU** | 0.0179 | 0.0151 | 0.0247 | 0.0188 | 0.0171 | 0.0175 | 0.0193 | 0.0169 | 0.0238 | 0.0207 | 0.0201 |
|  | **FIN** | 0.0252 | 0.0214 | 0.0293 | 0.0258 | 0.0242 | 0.0243 | 0.0258 | 0.0234 | 0.0294 | 0.0254 | 0.0221 |
|  | **GBR** | 0.0183 | 0.0154 | 0.0250 | 0.0191 | 0.0175 | 0.0178 | 0.0197 | 0.0172 | 0.0241 | 0.0210 | 0.0204 |
|  | **IBS** | 0.0148 | 0.0134 | 0.0245 | 0.0172 | 0.0153 | 0.0157 | 0.0180 | 0.0153 | 0.0223 | 0.0204 | 0.0201 |
|  | **TSI** | 0.0119 | 0.0105 | 0.0219 | 0.0137 | 0.0121 | 0.0125 | 0.0146 | 0.0124 | 0.0198 | 0.0178 | 0.0184 |
| **SAS** | **BEB** | 0.0334 | 0.0306 | 0.0251 | 0.0336 | 0.0342 | 0.0327 | 0.0313 | 0.0299 | 0.0295 | 0.0227 | 0.0210 |
|  | **GIH** | 0.0285 | 0.0261 | 0.0205 | 0.0276 | 0.0287 | 0.0273 | 0.0255 | 0.0248 | 0.0248 | 0.0183 | 0.0208 |
|  | **ITU** | 0.0323 | 0.0301 | 0.0234 | 0.0319 | 0.0329 | 0.0314 | 0.0296 | 0.0287 | 0.0279 | 0.0214 | 0.0233 |
|  | **PJL** | 0.0234 | 0.0209 | 0.0164 | 0.0226 | 0.0234 | 0.0221 | 0.0206 | 0.0198 | 0.0203 | 0.0141 | 0.0162 |
|  | **STU** | 0.0330 | 0.0308 | 0.0240 | 0.0328 | 0.0338 | 0.0323 | 0.0305 | 0.0295 | 0.0286 | 0.0221 | 0.0236 |
