# Supplementary material for: Distinct genetic variation and heterogeneity of the Iranian population
Source: PLoS Genet. 2019 Sep 24;15(9):e1008385. doi: 10.1371/journal.pgen.1008385 (PMC6759149; doi:10.1371/journal.pgen.1008385)
Supplement: S11 Table — (DOCX) [file pgen.1008385.s030.docx]

**Table S11. Pairwise identity-by-descent (IBD) sharing within Iranian ethnic groups.**

|  |  | **Number of pairs falling into IBD sharing classes (Proportion of pairs)** | | | | |
| --- | --- | --- | --- | --- | --- | --- |
| **Ethnic group**  **Iranian …** | **Number of pairs** | **Relatives  [2.125-26.0%]** | **1/4  [24.0-26.0%]** | **1/8  [11.5-13.5%]** | **1/16  [5.25-7.25%]** | **1/32  [2.125-4.124%]** |
| Arabs | 4560 | 47 (1.0%) | 1 (0.02%) | 0 | 1 (0.02%) | 39 (0.9%) |
| Azeris | 4851 | 76 (1.6%) | 0 | 0 | 0 | 75 (1.5%) |
| Baluchis | 4186 | 273 (6.5%) | 4 (0.10%) | 8 (0.19%) | 20 (0.48%) | 181 (4.3%) |
| Gilaks | 2775 | 1589 (57.3%) | 0 | 0 | 1 (0.04%) | 1547 (55.7%) |
| Kurds | 4656 | 1465 (31.5%) | 0 | 0 | 8 (0.17%) | 1443 (31.0%) |
| Lurs | 4753 | 631 (13.3%) | 1 (0.02%) | 0 | 2 (0.04%) | 604 (12.7%) |
| Mazanderanis | 3741 | 886 (23.7%) | 0 | 1 (0.03%) | 2 (0.05%) | 877 (23.4%) |
| Persians | 4465 | 133 (3.0%) | 0 | 1 (0.02%) | 4 (0.09%) | 122 (2.7%) |
| PG Islanders | 4095 | 424 (10.4%) | 0 | 2 (0.05%) | 44 (1.07%) | 274 (6.7%) |
| Sistanis | 4371 | 60 (1.4%) | 0 | 0 | 3 (0.07%) | 56 (1.3%) |
| Turkmen | 4656 | 218 (4.7%) | 0 | 1 (0.02%) | 8 (0.17%) | 200 (4.3%) |
